# Cobinamide is a strong and versatile antioxidant that overcomes oxidative stress in cells, flies, and diabetic mice

**DOI:** 10.1093/pnasnexus/pgac191

**Published:** 2022-09-14

**Authors:** Stephen Chang, John Tat, Shyamsundar Pal China, Hema Kalyanaraman, Shunhui Zhuang, Adriano Chan, Cassandra Lai, Zoran Radic, Engy A Abdel-Rahman, Darren E Casteel, Renate B Pilz, Sameh S Ali, Gerry R Boss

**Affiliations:** Department of Medicine, University of California, San Diego, La Jolla, CA 92093, USA; Department of Medicine, University of California, San Diego, La Jolla, CA 92093, USA; Department of Medicine, University of California, San Diego, La Jolla, CA 92093, USA; Department of Medicine, University of California, San Diego, La Jolla, CA 92093, USA; Department of Medicine, University of California, San Diego, La Jolla, CA 92093, USA; Department of Medicine, University of California, San Diego, La Jolla, CA 92093, USA; Department of Medicine, University of California, San Diego, La Jolla, CA 92093, USA; Skaggs School of Pharmacy and Pharmaceutical Sciences, University of California, San Diego, La Jolla, CA 92093, USA; Tumor Biology Research Program, Children’s Cancer Hospital, Cairo 57357, Egypt; Pharmacology Department, Faculty of Medicine, Assuit University, Assuit 71515, Egypt; Department of Medicine, University of California, San Diego, La Jolla, CA 92093, USA; Department of Medicine, University of California, San Diego, La Jolla, CA 92093, USA; Tumor Biology Research Program, Children’s Cancer Hospital, Cairo 57357, Egypt; Department of Medicine, University of California, San Diego, La Jolla, CA 92093, USA

**Keywords:** antioxidant, cobinamide, diabetes, oxidative stress

## Abstract

Increased oxidative stress underlies a variety of diseases, including diabetes. Here, we show that the cobalamin/vitamin B_12_ analog cobinamide is a strong and multifaceted antioxidant, neutralizing superoxide, hydrogen peroxide, and peroxynitrite, with apparent rate constants of 1.9 × 10^8^, 3.7 × 10^4^, and 6.3 × 10^6^ M^−1^ s^−1^, respectively, for cobinamide with the cobalt in the +2 oxidation state. Cobinamide with the cobalt in the +3 oxidation state yielded apparent rate constants of 1.1 × 10^8^ and 8.0 × 10^2^ M^−1^ s^−1^ for superoxide and hydrogen peroxide, respectively. In mammalian cells and *Drosophila melanogaster*, cobinamide outperformed cobalamin and two well-known antioxidants, imisopasem manganese and manganese(III)tetrakis(4-benzoic acid)porphyrin, in reducing oxidative stress as evidenced by: (i) decreased mitochondrial superoxide and return of the mitochondrial membrane potential in rotenone- and antimycin A-exposed H9c2 rat cardiomyocytes; (ii) reduced JNK phosphorylation in hydrogen-peroxide-treated H9c2 cells; (iii) increased growth in paraquat-exposed COS-7 fibroblasts; and (iv) improved survival in paraquat-treated flies. In diabetic mice, cobinamide administered in the animals’ drinking water completely prevented an increase in lipid and protein oxidation, DNA damage, and fibrosis in the heart. Cobinamide is a promising new antioxidant that has potential use in diseases with heightened oxidative stress.

Significance Statement An increase in oxidative stress likely plays a causal role in many different diseases, but antioxidant drugs have not had a convincingly positive effect. This may be because current antioxidants are insufficiently potent or neutralize a limited number of reactive oxygen or reactive nitrogen species. We found that the vitamin B_12_ analog cobinamide is a potent and wide-ranging antioxidant that neutralizes several major reactive oxygen and nitrogen species. It was highly effective at reducing oxidative stress in cultured mammalian cells, fruit flies, and diabetic mice, and was considerably more effective in cells and flies than several well-known antioxidants. Cobinamide could potentially be used to treat a variety of diseases with increased oxidative stress.

## Introduction

**Fig. 1.**
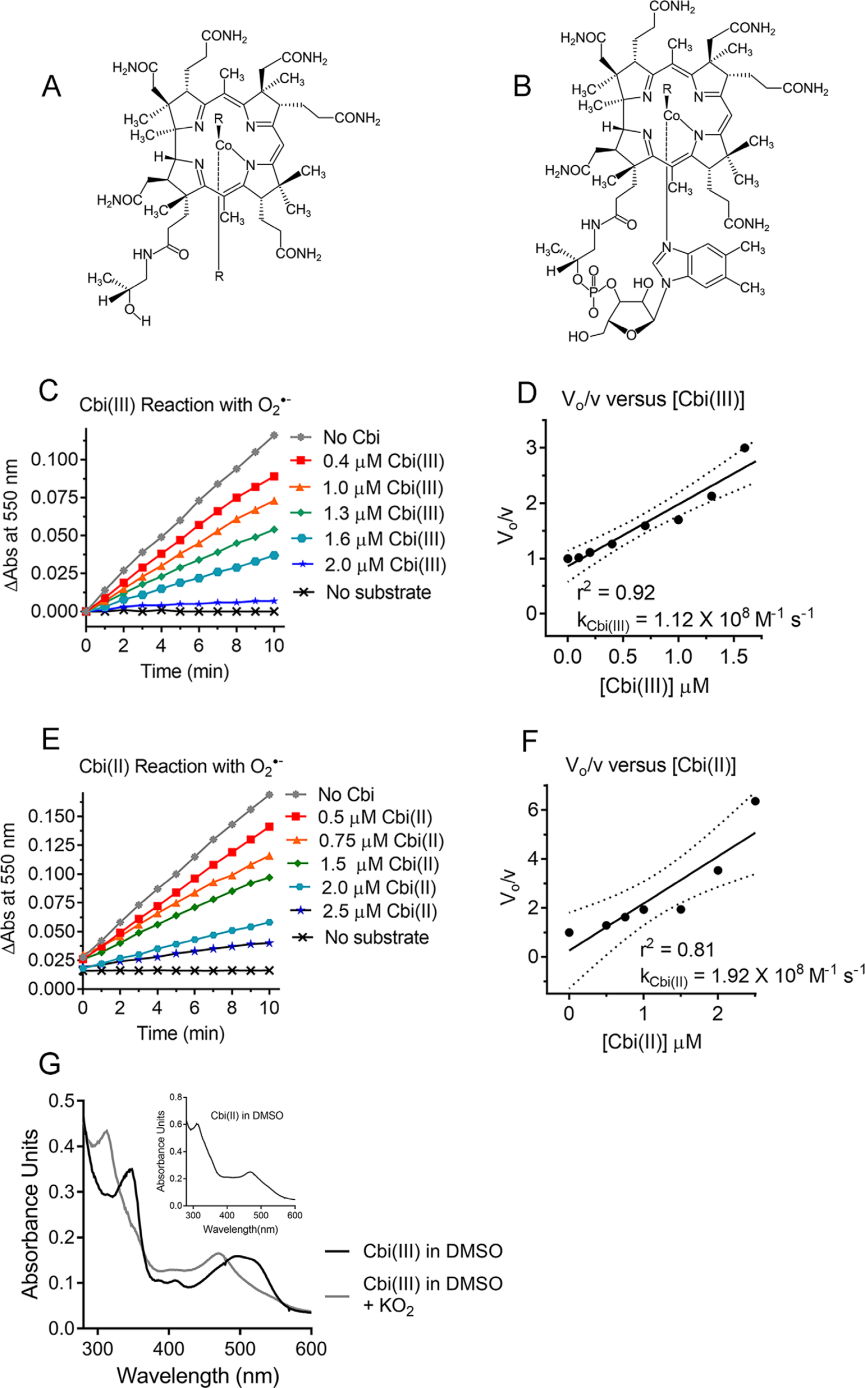
Cobinamide functions as a superoxide dismutase (SOD) mimetic. (A, B) Chemical structures of aquohydroxo-cobinamide (A) and hydroxo-cobalamin (B). (C to F) Increasing concentrations of Cbi(III) (C) or Cbi(II) (E) were incubated in a hypoxanthine–xanthine oxidase-cytochrome c-catalase system for measuring O_2_·^−^, with the change in absorbance at 550 nm (ΔAbs) plotted versus time. A no substrate (no hypoxanthine) control was included. (D, F) The calculated rate of change of absorbance at 550 nm in the absence of Cbi(III) or Cbi(II) (V_o_) divided by the calculated rate in their presence (v) is plotted against the Cbi(III) (D) or Cbi(II) (F) concentration, respectively. The slope of the fitted line was used to calculate the apparent rate constant according to the following equation: k_Cbi_ = slope X k_cytochrome c_ X [cytochrome c], where k_cytochrome c_ = 1.4 × 10^6^ M^−1^ s^−1^ (70) and [cytochrome c] = 70 µM. The data shown are the mean of duplicate samples from one experiment, with similar results found in two additional experiments. Dotted lines show 95% CIs. (G) A 20 µM solution of Cbi(III) in DMSO was scanned from 280 to 600 nm yielding the spectrum shown as a black line. An equimolar amount of KO_2_ dissolved in DMSO was added and the solution was scanned immediately, yielding the gray line. The inset shows Cbi(II) in DMSO generated by ascorbate reduction of Cbi(III). The experiment was conducted three times with identical results.

Cobinamide is a late precursor in cobalamin (vitamin B_12_) biosynthesis by microorganisms (1). It lacks the dimethylbenzimidazole ligand of cobalamin, which is replaced by water in aqueous solutions (Fig. 1A and B; please see the “Methods” section for cobinamide nomenclature). This imparts several important chemical differences between cobinamide and cobalamin. First, cobinamide can bind two ligands instead of only one. Second, the bulky dimethylbenzimidazole ligand of cobalamin reduces the affinity of the cobalt for ligands in the *trans* position (2). Third, cobinamide is considerably more water soluble than cobalamin, and by lacking the relatively labile phosphodiester group, is more stable in aqueous solutions than cobalamin (3, 4). And fourth, relevant to the results reported here, the cobalt in cobinamide is more easily reduced than the cobalt in cobalamin, likely due to the considerably lower electron-donating ability of water compared to dimethylbenzimidazole: the standard potential for the reduction of the cobalt from the +3 oxidation state to the +2 oxidation state in cobalamin is −40 mV compared to +270 mV for the cobalt in cobinamide (5, 6).

We have shown in numerous in-vitro studies in cultured cells and in-vivo studies in fruit flies, mice, rabbits, and pigs that cobinamide is an excellent cyanide and hydrogen sulfide antidote, much better than cobalamin, an approved treatment for cyanide poisoning (7–11). Cobinamide and cobalamin both serve as cyanide and sulfide scavengers, but due to cobinamide’s higher affinity for ligands, cobinamide is a more efficient scavenger (2). Cyanide and sulfide inhibit cytochrome c oxidase in complex IV of the mitochondrial electron transport chain, thereby generating superoxide (O_2_·^−^) and inducing intracellular oxidative stress (10, 12). Cobalamin with the cobalt in the +2 oxidation state reacts with O_2_·^−^, peroxynitrite (ONOO^−^), and hypochlorous acid, and in both the +3 and +2 oxidation states with hydrogen peroxide (H_2_O_2_) and the carbonate radical anion (CO_3_·^−^) (13–18). Consistent with these in-vitro data, cobalamin functions as an antioxidant in several cell and animal models (19–23). We have shown that cobinamide reduces oxidative stress in sulfide-poisoned mice, suggesting that part of cobinamide’s and cobalamin’s activity against cyanide and hydrogen sulfide is due to their antioxidant properties (10).

Here, we determined reaction rates of cobinamide in the +3 and +2 oxidation states [cobinamide(III) and cobinamide(II), abbreviated as Cbi(III) and Cbi(II), respectively] with O_2_·^−^ and H_2_O_2_ and reaction rates of Cbi(II) with ONOO^−^. We compared the rates to those published for cobalamin and the potent superoxide dismutase (SOD) mimetic imisopasem manganese (M40403) and the membrane-permeable SOD mimetic and ONOO^−^ scavenger MnTBAP [manganese(III)tetrakis(4-benzoic acid)porphyrin] (24–28). Like cobalamin, the latter two compounds have been shown to serve as antioxidants in cells and animals (26, 27, 29–32). We found that the reaction rates of Cbi(III) and Cbi(II) with the oxidizing species were generally comparable to or substantially higher than those for the other three agents, and that cobinamide was considerably better than all three agents at reducing oxidative stress in mammalian cells and fruit flies. Furthermore, cobinamide overcame lipid and protein oxidation, DNA damage, and excessive fibrosis in the hearts of diabetic mice when administered in drinking water at a dose well tolerated by the mice.

## Results

### Cobinamide functions as a SOD mimetic

The metal centers of SOD are cyclically reduced and oxidized, in the process converting O_2_·^−^ to O_2_ and H_2_O_2_ (33). Cbi(III) is relatively easily reduced to Cbi(II) and the latter can reoxidize to Cbi(III) (5, 6). This suggests cobinamide could serve as an SOD mimetic according to the following two reactions:

(1) $$\begin{equation*}
{\rm{Cbi}}\left( {{\rm{III}}} \right) + {{\rm{O}}}_2{\cdot}^ - \to {\rm{Cbi}}\left( {{\rm{II}}} \right) + {{\rm{O}}}_2,
\end{equation*}
$$

(2) $$\begin{equation*}
{\rm{Cbi}}\left( {{\rm{II}}} \right) + {{\rm{O}}}_2{\cdot}^ - + 2\,{{\rm{H}}}^ + \to {\rm{Cbi}}\left( {{\rm{III}}} \right) + {{\rm{H}}}_2{{\rm{O}}}_2
.
\end{equation*}
$$

We found that both Cbi(III) and Cbi(II) reacted readily with O_2_·^−^, as measured in a hypoxanthine–xanthine oxidase-cytochrome c reduction system, yielding apparent rate constants of 1.12 and 1.92 × 10^8^ M^−1^ s^−1^ for the Cbi(III) and Cbi(II) reactions, respectively (Fig. 1C to F, Table 1). We included catalase in the reaction mixture, because the hypoxanthine–xanthine oxidase system also generates H_2_O_2_ and the latter can oxidize the ferro-cytochrome c product back to ferric-cytochrome c, thereby interfering with the kinetic measurement (34).

**Table 1.** Reaction rates of cobinamide (Cbi), cobalamin, imisopasem, and MnTBAP with reactive oxygen and nitrogen species.

**Compound**	**Superoxide (O_2_·^−^)(^a^)**	**Hydrogen peroxide (H_2_O_2_)(^b^)**	**Peroxynitrite (ONOO^−^)**	**Nitric oxide (·NO)**
Cbi(III)	1.1 × 10^8^ (^c^)	8.0 × 10^2^ (^c^)	Not studied	2.4 × 10^8^ (51)
Cbi(II)	1.9 × 10^8^ (^c^)	3.7 × 10^4^ (^c^)	6.3 × 10^6^ (^c^)	K_a:_ 1.3 × 10^10^ (51)
Cobalamin(III)	9.4 × 10^6^ (^c^)	4.5 × 10^6^ (17)	^(^^d^^)^	No activity (51,71)
Cobalamin(II)	3.8 × 10^8^ (13)	1.4 × 10^2^ (16)	3.7 × 10^5^ (14)	K_a:_ 1.5 × 10^8^ (51)
Imisopasem	1.6 × 10^7^ (24)	8.2 (25)	No activity (26)	No activity (26)
MnTBAP	∼ 4.0 (54)	5.8 (25)	∼ 1 × 10^5^ (27)	No activity (28)

Reactions of the indicated compounds with O_2_·^−^, ONOO^−^, and ·NO are all second order reactions with rate constants in units of M^−1^ s^−1^. The reactions of Cbi(III) and Cbi(II) with H_2_O_2_ are third order with rate constant units of M^−2^ s^−1^. Cbi(II) and cobalamin(II) bind nitric oxide, so a K_a_ is shown in units of M^−1^. For the new data presented in this study, rate constants were measured for O_2_·^−^ using a hypoxanthine–xanthine oxidase-cytochrome c system, for H_2_O_2_ using a H_2_O_2_-specific sensor (World Precision Instruments), and for ONOO^−^ using a stopped-flow instrument. Published rate constants for Cu/Zn and Mn SODs and catalase are shown for reference.^a, b^ Letters and numbers in parenthesis refer to footnotes and references, respectively.

a Mammalian Cu/Zn SODs 1 and 3: 2–6 × 10^9^ (33, 72); mitochondrial Mn SOD 2: 7–10 × 10^8^ (33, 73).

b Mammalian catalase: 1.5 × 10^6^ (25).

c New data presented in this study.

d No published data found.

We additionally measured Cbi(III) reaction with O_2_·^−^ using the spin-trap 5,5-dimethyl-1-pyrroline N-oxide (DMPO), which yields a distinctive electron paramagnetic resonance (EPR) spectrum on reacting with O_2_·^−^. We found that submicromolar concentrations of cobinamide reduced the EPR signal in a dose-dependent fashion, yielding an apparent rate constant of 7.5 × 10^7^ M^−1^ s^−1^ for the reaction of Cbi(III) with O_2_·^−^ (Fig. S1A and B). This value is within experimental error of the value of 1.12 × 10^8^ M^−1^ s^−1^ found in the cytochrome c reduction system.

To study the mechanisms of the reactions and provide evidence for equations (1) and (2), we followed the reactions spectrophotometrically and by visible color change. We studied the reduction of Cbi(III) to Cbi(II) in dimethyl sulfoxide (DMSO); O_2_·^−^ is relatively stable in aprotic solvents and the lack of hydrogen ions prevents reoxidation of Cbi(II) by O_2_·^−^ (35). Thus, we were able to focus solely on the reaction of Cbi(III) with O_2_·^−^, and found that O_2_·^−^ fully reduced Cbi(III) to Cbi(II) (Fig. 1G). To study O_2_·^−^ oxidation of Cbi(II) to Cbi(III), we added O_2_·^−^ in DMSO to an aqueous solution of Cbi(II). Cbi(II) was rapidly oxidized to Cbi(III) and then largely reduced back to Cbi(II). We demonstrated this by recording a video at 30 frames per second (see the “Supplementary Material” section). The video shows that Cbi(II), which is yellow, is oxidized to Cbi(III), which is pink, and then the color returns back towards yellow; the wells on either side of the test well contain Cbi(II) on the left and Cbi(III) on the right for comparison. Thus, it appears cobinamide can serve as an SOD mimetic by cycling between Cbi(III) and Cbi(II); the reaction rates of Cbi(III) and Cbi(II) with O_2_·^−^ are about 10 and many orders of magnitude faster than the reaction rates of imisopasem and MnTBAP with O_2_·^−^, respectively (Table 1).

Cobalamin(II) has been shown to react with O_2_·^−^ at a rate two times faster than we found for Cbi(II) [(13) and Table 1], but to our knowledge, the reaction of cobalamin(III) with O_2_·^−^ has not been reported. We found that cobalamin(III) reacts with O_2_·^−^, but at a rate less than one-tenth that of Cbi(III) (Figure S1C, D, Table 1).

### Cobinamide functions as a catalase mimetic

Like SOD, catalase has a metal (iron) center that is alternately reduced and oxidized during the conversion of H_2_O_2_ to H_2_O and O_2_ (36). We hypothesized that cobinamide could function as a catalase mimetic according to reactions (3) and (4):

(3) $$\begin{equation*}
2\,{\rm{Cbi}}\left( {{\rm{III}}} \right) + {{\rm{H}}}_2{{\rm{O}}}_2 \to 2\,{\rm{Cbi}}\left( {{\rm{II}}} \right) + {{\rm{O}}}_2 + 2\,{{\rm{H}}}^ +
.
\end{equation*}
$$

(4) $$\begin{equation*}
2\,{\rm{Cbi}}\left( {{\rm{II}}} \right)\ + \ {{\rm{H}}}_2{{\rm{O}}}_2 + {\rm{2}}\,{{\rm{H}}}^ + \to 2\,{\rm{Cbi}}\left( {{\rm{III}}} \right) + 2\,{{\rm{H}}}_2{\rm{O}}.
\end{equation*}
$$

**Fig. 2.**
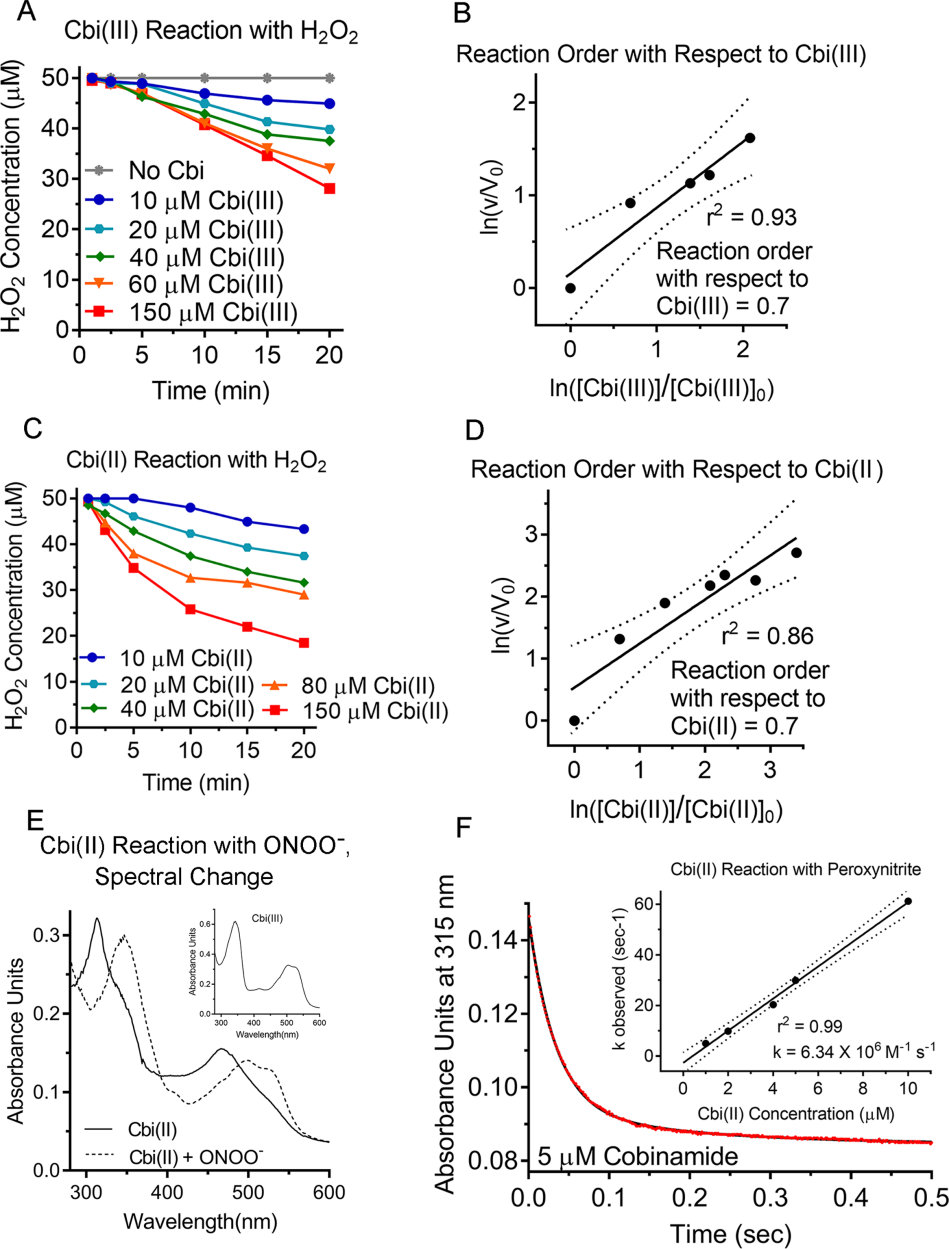
Cobinamide functions as a catalase mimetic and reacts with peroxynitrite. (A to D) Hydrogen peroxide (final concentration 50 µM) was added to solutions containing increasing concentrations of Cbi(III) (A) or Cbi(II) (C), and the H_2_O_2_ concentration was measured over time using a H_2_O_2_-specific electrode. The log of the velocity in the presence of Cbi(III) or Cbi(II) (v) over the velocity in their absence (V_o_) is plotted against the log of their concentrations at 20 min over their concentrations at zero time (B and D, respectively). These plots yield a reaction order with respect to Cbi(III) and Cbi(II) of 0.7. The experiments were repeated three times with similar results. Dotted lines show 95% CIs. (E) Adding an equimolar amount of peroxynitrite to a 25 µM Cbi(II) solution in 50 mM sodium phosphate, pH 11 immediately changed the UV-visible spectrum to that of Cbi(III) [inset shows the spectrum of authentic Cbi(III) at pH 11]. The Cbi(III) spectra are slightly different from that shown in Fig. S2E because at pH 11, dihydroxo-cobinamide is generated, whereas at pH 7.1, aquohydroxo-cobinamide is the predominant species. The spectra of aquohydroxo- and dihydroxo-cobinamide are known to be different (65). (F) Varying concentrations of Cbi(II) from 1 to 10 µM were mixed with 10 µM peroxynitrite in a stopped-flow instrument, and absorption at 315 nm was monitored for 0.5 s. The results for 5 µM Cbi(II) are shown; red circles are the observed data, and the black line is a two-phase nonlinear regression curve generated by Prism 7.04 software. The inset shows a plot of *k*_observed_ versus the Cbi(II) concentration, yielding an apparent rate constant for the reaction between Cbi(II) and peroxynitrite of 6.34 × 10^6^ M^−1^ s^−1^. The experiment was repeated three times with similar results. Dotted lines show 95% CIs.

We found that both Cbi(III) and Cbi(II) react with H_2_O_2_, and that the reaction was essentially first order with respect to Cbi(III) and Cbi(II) (Fig. 2A to D), and second order with respect to H_2_O_2_ for both cobinamide species (Fig. S2A to D). Using these data, we calculated the apparent rate constants to be 8.00 × 10^2^ and 3.68 × 10^4^ M^−2^ s^−1^ for Cbi(III) and Cbi(II), respectively (Table 1). Because the overall rate constants are third order, it is not possible to compare them strictly with published rate constants for the reaction of H_2_O_2_ with imisopasem, MnTBAP, and cobalamin shown in Table 1. To assess if cobinamide was acting via equations (3) and (4), we performed similar experiments to those done with O_2_·^−^: we showed that the reactions of H_2_O_2_ with Cbi(III) and Cbi(II) generated in a concentration and time-dependent fashion largely Cbi(II) and Cbi(III), respectively, although the final products were intermediate species (Fig. S2E and F). Thus, cobinamide appears to function as a catalase mimetic.

### Cbi(II) reacts with peroxynitrite

Peroxynitrite (ONOO^−^) is a potent oxidizing agent that has been shown to oxidize cobalamin(II) to cobalamin(III) (14, 37). We found that ONOO^−^ oxidizes Cbi(II) rapidly to Cbi(III) (Fig. 2E). We then studied the rate of the reaction in stopped-flow experiments, and found an apparent rate constant of 6.34 × 10^6^ M^−1^ s^−1^, or more than 10- and 50-fold higher than the reactions of cobalamin(II) and MnTBAP with ONOO^−^, respectively (Fig. 2F, Table 1).

### Cobinamide rescues cells from oxidative stress, and is superior to cobalamin, imisopasem, and MnTBAP

**Fig. 3.**
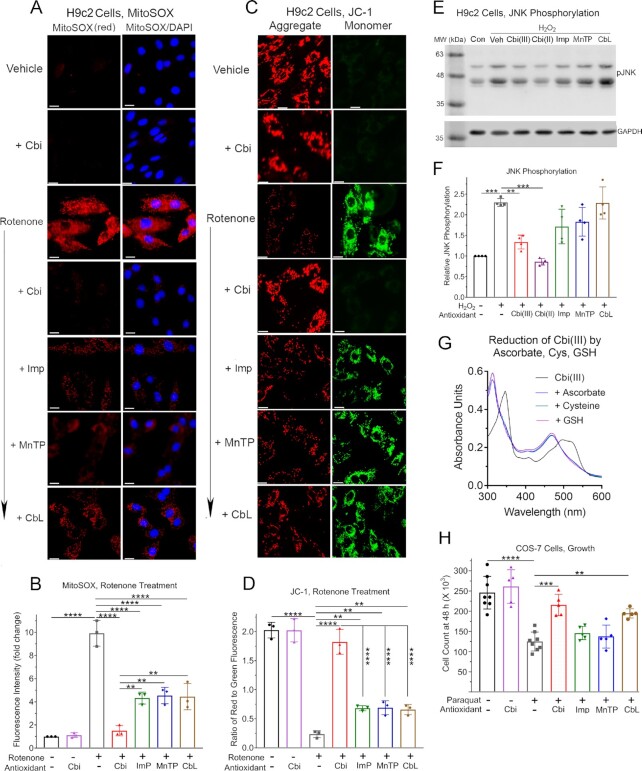
Cobinamide rescues cells from oxidative stress, and is superior to other antioxidants; reduction of Cbi(iii) by ascorbate, cysteine, and reduced glutathione. (A, B) H9c2 cells were incubated for 30 min with vehicle, 2.5 µM cobinamide, or 5 µM rotenone, the latter in the absence or presence of 2.5 µM cobinamide, imisopasem, MnTBAP, or cobalamin; 5 µM MitoSOX was added during the last 10 min. The cells were incubated with 1 mg/ml DAPI for 3 min to counterstain nuclei, and then visualized under a fluorescence microscope. (A) Equal-sized representative areas for each condition are shown for MitoSOX staining (red fluorescence) and the same area showing merged MitoSOX and DAPI staining. (B) The amount of red fluorescence was quantified by Image J analysis. Data are the mean ± SD of three independent experiments; in each experiment two separate equal-sized areas were analyzed containing ∼75 cells per area. (C, D) H9c2 cells were incubated for 10 min with 10 µM JC-1, washed once with PBS, and then incubated for 30 min with vehicle, 2.5 µM cobinamide, or 5 µM rotenone in the absence or presence of 2.5 µM of the indicated drugs as described in Panels A, B. (C) Cells were visualized under a fluorescence microscope and equal-sized representative areas of each condition are shown, with red and green fluorescence shown for the same area. (D) The amount of red and green fluorescence was quantified by Image J analysis and the red to green ratio was calculated. The slides were analyzed by an operator who was blinded to the specific conditions. Data are the mean ± SD of three independent experiments; in each experiment two separate equal-sized areas were analyzed containing ∼75 cells per area. (E, F) H9c2 cells were incubated for 30 min in the absence of any addition (control, Con) or with 100 µM H_2_O_2_ in the absence (vehicle, Veh) or presence of 100 µM of the indicated drugs. The cells were extracted in a SDS-based buffer, proteins were resolved by PAGE, and phospho-JNK(Thr^183^/Tyr^185^) (pJNK) was identified by immunoblotting (upper blot). The two isoforms of pJNK have observed molecular weights of 46 and 54 kDa. The blot was stripped and reprobed with an antibody against GAPDH (lower blot). (E) Representative blots are shown. The two isoforms of JNK have observed molecular weights of 46 and 54 kDa. (F) Blots from four independent experiments were analyzed by densitometric scanning (Li-Cor Odyssey software) in a range where band intensity was linear. The ratio of the pJNK band to GAPDH was calculated, and the results normalized to control untreated cells. The data are the mean ± SD of the four experiments. (G) The UV-visible spectrum of Cbi(III) in 50 mM potassium phosphate buffer, pH 7.4 was recorded before (black line) and immediately after adding a three-fold molar excess of ascorbate (blue line), cysteine (teal line), or reduced glutathione (GSH, pink line). (H) COS-7 cells were incubated for 3 h with 1 mM paraquat, in the absence or presence of 100 µM of the indicated drugs. The cells were counted 48 h later using a hemocytometer. The data are the mean ± SD of at least five experiments per condition. Veh, vehicle; Cbi, cobinamide; Imp, imisopasem; MnTP, MnTBAP; CbL, cobalamin; GSH, reduced glutathione. White scale bar in A and C is 10 µM in length. The data were analyzed by a one-way ANOVA (for Panels B, D, and H, *P* < 0.0001 and for Panel F, *P* = 0.0068) followed by Tukey’s multiple comparison test of all conditions; **, ***, and **** indicate *P* < 0.01, 0.001, and 0.0001, respectively, for the indicated paired comparisons. In Panels B and D, comparison of vehicle-treated cells to cells treated with cobinamide in the absence or presence of rotenone was not significant, whereas comparison of vehicle-treated cells to rotenone-exposed cells treated with imisopasem, MnTBAP, or cobalamin was significant, with the *P*-values at least <0.001. In Panel F, comparison of control untreated cells to cells receiving H_2_O_2_ and Cbi(III) or Cbi(II) was not significant.

To assess if cobinamide reduces oxidative stress in cells and to compare its in-vivo efficacy to that of cobalamin, imisopasem, and MnTBAP, we performed several sets of experiments. First, we evaluated if cobinamide reduces mitochondrial-generated O_2_·^−^ using the fluorescent probe MitoSOX Red. We treated H9c2 rat embryonal cardiomyocytes with rotenone and antimycin A, inhibitors of complex I and III, respectively, to increase mitochondrial O_2_·^−^. We found minimal fluorescence in vehicle-treated cells with no change on adding cobinamide, but a marked increase in fluorescence in rotenone- and antimycin A-treated cells (Fig. 3A and B show rotenone-treated cells and Fig. S3A and B show antimycin A-treated cells; note that the fluorescence is largely extranuclear). As described in the “Methods” section and shown in Table S1, we found that rotenone and antimycin A increased 2-hydroxy-mitoethidium without increasing mitoethidium, indicating that the two drugs mainly increased O_2_·^−^and not other species that oxidized MitoSOX. Cobinamide markedly reduced fluorescence in rotenone- and antimycin A-treated cells to a signal indistinguishable from that found in vehicle-treated cells (Fig. 3A and B and Fig. S3A and B). Imisopasem, MnTBAP, and cobalamin also reduced the fluorescent signal substantially in rotenone- and antimycin A-treated cells compared to vehicle-treated cells, but the signal remained significantly higher than in cells receiving cobinamide (Fig. 3A and B and Fig. S3A and B; all four drugs were present at 2.5 µM). Cobinamide’s beneficial effect was not due to directly binding, and thereby scavenging, rotenone or antimycin A, because we found no change in the UV-visible spectrum of cobinamide in the presence of an eight-fold excess of rotenone or antimycin A (Fig. S3C and D, respectively). Monitoring the UV-visible spectrum of cobinamide is a sensitive means to assess ligand binding (38). Nor did cobinamide appear to affect mitochondrial mass, because alone it did not significantly change the amount of 2-hydroxy-mitoethidum or mitoethidium (Table S1).

Increased mitochondrial O_2_·^−^ by rotenone and antimycin A would be expected to reduce the mitochondrial membrane potential (ΔΨm), due in part to activation of uncoupling proteins (39). We used the dye JC-1 to assess ΔΨm: JC-1 aggregates and shows red fluorescence in mitochondria with high ΔΨm and stays as a monomer with green fluorescence in mitochondria with low ΔΨm (40). We observed a high red to green fluorescence ratio in vehicle-treated H9c2 cells with no change on adding cobinamide, indicating a relatively high ΔΨm in the cells (Fig. 3C and D and Fig. S3E and F). Rotenone and antimycin A caused an almost complete loss of red fluorescence and a striking increase in green fluorescence in the cells, indicating a marked decrease in ΔΨm (Fig. 3C and D for rotenone-treated cells and Fig. S3E and F for antimycin A-treated cells). Cobinamide restored the ratio of red to green fluorescence in rotenone- and antimycin A-treated cells to a value indistinguishable from that in vehicle-treated cells, indicating a return of ΔΨm (Fig. 3C and D and Fig. S3E and F). Imisopasem, MnTBAP, and cobalamin all showed modest degrees of recovery of red to green fluorescence in rotenone- and antimycin A-treated cells; as with the measurement of O_2_·, the three drugs did not return fluorescence to values found in vehicle-treated cells and cobinamide was significantly better than the three drugs (Fig. 3C and D and Fig. S3E and F; the four drugs were present at 2.5 µM).

Next, we tested the effect of cobinamide and the comparator drugs on hydrogen peroxide-induced oxidative stress. We treated H9c2 cells with H_2_O_2_ and assessed phosphorylation/activation of Jun N-terminal kinase [JNK, also known as stress-activated protein kinase (SAPK)]; JNK is activated downstream of a variety of reactive oxygen species, including H_2_O_2_ (10). We found that 30 min of H_2_O_2_ treatment increased JNK phosphorylation 2.4-fold, and that both Cbi(III) and Cbi(II) reduced JNK phosphorylation to a level that was indistinguishable from vehicle-treated cells (Fig. 3E and F). We had to use higher cobinamide concentrations (100 µM) in these studies than in the rotenone and antimycin A studies, possibly because cobinamide reacts less readily with H_2_O_2_ than with O_2_·^−^ (Table 1). Imisopasem and MnTBAP had small non-significant effects and cobalamin had no effect on JNK phosphorylation (Fig. 3E and F; the drugs were at 100 µM). The cobalamin was in the +3 oxidation state, i.e. cobalamin(III); we could not test cobalamin(II) because in the presence of oxygen, it rapidly oxidized back to cobalamin(III). In the absence of H_2_O_2_, neither Cbi(III), Cbi(II) nor the other three drugs affected JNK phosphorylation or total cellular JNK (Fig. S3G). As part of these studies, we found that Cbi(III) is readily reduced to Cbi(II) by ascorbate, cysteine, and reduced glutathione (GSH) under physiological conditions (Fig. 3G) [previous workers have also found that GSH reduces Cbi(III) to Cbi(II) (41)]. The implications of the facile reduction of Cbi(III) to Cbi(II) will be considered in the “Discussion” section.

Finally, we tested if cobinamide could alleviate the effects of oxidative stress induced by yet another mechanism in a different cell type, and used paraquat-treated COS-7 cells. Paraquat generates O_2_·^−^ through a redox cycling mechanism and increases both intracellular O_2_·^−^ and H_2_O_2_ (42). We found that exposing COS-7 cells for 3 h to 1 mM paraquat reduced cell growth by ∼50%, when measured 48 h later (Fig. 3H). Cobinamide alone had no effect on cell growth and when added to paraquat-treated cells, it restored growth to ∼80% of the control value (Fig. 3H). Cobalamin also improved cell growth, but it was not as effective as cobinamide, and neither imisopasem nor MnTBAP had an effect (Fig. 3H). As in the studies with rotenone and antimycin A, cobinamide did not appear to be acting by directly binding and scavenging paraquat, because we found no change in the UV-visible spectrum of cobinamide at paraquat concentrations up to eight times higher than that of cobinamide (Fig. S3H).

### Cobinamide rescues flies from paraquat poisoning

**Fig. 4.**
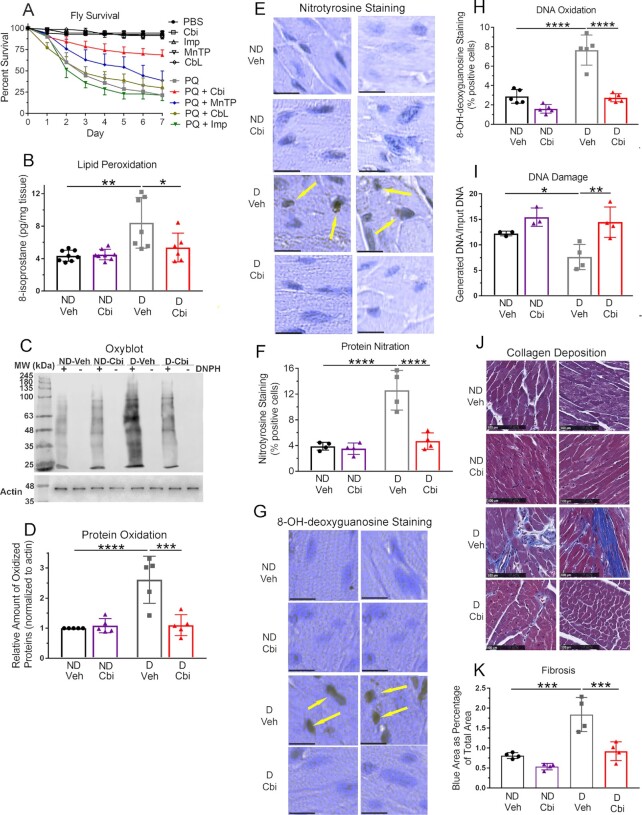
Cobinamide rescues flies from paraquat poisoning and prevents abnormal lipid and protein oxidation, DNA damage, and fibrosis in the hearts of diabetic mice. (A) At time zero, 10 fruit flies (*D. melanogaster*) were transferred to vials with food to which either phosphate-buffered saline (PBS), 0.8 mM of the indicated drugs, 20 mM paraquat (PQ), or 20 mM paraquat with 0.8 mM of the indicated drugs had been added. The number of live flies was counted each day for 7 d. The experiment was repeated seven times, for a total of 70 flies per condition. The % surviving flies was calculated; error bars represent SDs and are shown in one direction only for sake of clarity. (B to K) Twenty-week–old male C57BL/6NHsd mice were injected with saline or streptozotocin, and 13 d later, the mice were randomized to receive plain drinking water or 1 mM histidyl-cobinamide in the drinking water. After 3 months, they were euthanized and their hearts were flash frozen in liquid nitrogen or fixed in paraformaldehyde. Initially, each group had eight mice, but at the time of euthanasia, three of the streptozotocin-injected mice were found not to be diabetic (one mouse that did not receive cobinamide and two mice that did receive cobinamide); they were excluded from all analyses. The amount of 8-isoprostane (B) was measured by ELISA, protein oxidation (C, D) was assessed by immunoblotting, and nitrotyrosine content (E, F) was assessed by immunohistochemistry. DNA damage was assessed by 8-OH-deoxyguanosine staining (G, H) and a long amplicon qPCR-based assay (I). Cardiac fibrosis (J, K) was assessed by Mallory trichrome staining, which stains collagen blue. In Panels E, G, and J, the two adjacent squares are representative areas from two separate mice; the scale bars in Panels E and G are 10 µM and the bar in Panel J is 100 µM. The yellow arrows in Panels E and G indicate positively stained cells. For Panels F and H, at least 100 cells from each sample were visualized; the data shown are the % of cells that stained positive. For Panels C, D, and I, frozen heart samples were selected randomly from each of the four groups and were analyzed. For Panel D, blots were analyzed by densitometric scanning using Li-Cor Odyssey software, with the density of each lane normalized to the actin band. For Panel I, DNA was extracted and used as a template for PCR of an 8.7 kb fragment of the β-globin gene. Any DNA injury such as modified bases or strand breaks inhibit DNA synthesis, with the data expressed as the amount of DNA generated over the amount of input DNA. For Panel K, the area of blue staining within the total cross-section of the cardiac apex was measured using Image-Pro Premier software. The data are expressed as the % blue area within the total heart section and are the mean ± SD of hearts from four mice per condition. For Panels F, H, and K, slides from four to five mice from each of the four groups were selected randomly and analyzed by an operator blinded to the specific conditions. Cbi, cobinamide in Panel A and histidyl-cobinamide in Panels B to K; Imp, imisopasem; MnTP, MnTBAP; CbL, cobalamin; DNPH, 2,4-dinitrophenylhydrazine; ND, nondiabetic; D, diabetic; Veh, vehicle. The data were analyzed by a two-way ANOVA (interaction, *P* < 0.05 for all data shown in bar graphs) followed by Sidak’s multiple comparison test; *, **, ***, and **** indicate *P* < 0.05, 0.01, 0.001, and 0.0001, respectively, for the indicated paired comparisons. In all cases, comparison of the nondiabetic vehicle versus nondiabetic cobinamide groups and comparison of nondiabetic cobinamide versus diabetic cobinamide groups was not significant.

We next wanted to test cobinamide in a whole animal, and chose *Drosophila melanogaster* for our initial studies, increasing oxidative stress in the flies by administering paraquat in their food. We again compared cobinamide to imisopasem, MnTBAP, and hydroxocobalamin, all administered in food. We found that 0.8 mM of the drugs had no effect on the flies (Fig. 4A). Previous workers have found that *D. melanogaster* tolerate relatively high paraquat concentrations (43), and we found that 20 mM paraquat was required to yield >75% mortality over 7 d (Fig. 4A; *P* < 0.0001 by both Mantel-Cox log rank test and area under the curve analysis for comparison between untreated and paraquat-treated flies). Providing 0.8 mM cobinamide with the paraquat reduced mortality to <30%, while 0.8 mM MnTBAP had a lesser effect and 0.8 mM imisopasem and cobalamin had no significant effect (Fig. 4A; for cobinamide, *P* < 0.0001 by both log rank test and area under the curve analysis, and for MnTBAP, *P* < 0.01 by log rank test and < 0.0001 by area under the curve analysis for comparison to flies exposed to paraquat in the absence of drugs).

### Cobinamide prevents abnormal lipid and protein oxidation, DNA damage, and fibrosis in the hearts of diabetic mice

In the final set of experiments, we asked if cobinamide could reduce oxidative stress in a mammal exhibiting a common human disease. We chose diabetes in mice and examined the heart: diabetes leads to increased oxidative stress in many organs, including the heart where the increased oxidative stress causally contributes to a well-described cardiomyopathy (44, 45). We generated type I diabetic mice using streptozotocin, and then provided cobinamide in the drinking water for 3 months. At the time of euthanasia, the plasma cobinamide concentration in mice that had received cobinamide in their drinking water was 0.11 ± 0.07 µM for nondiabetic mice and 0.55 ± 0.36 µM for diabetic mice (Fig. S4A; sufficient plasma was available for four nondiabetic and four diabetic mice). The difference between the two groups was not significant, but the higher values in the diabetic mice were likely due to their increased water consumption. This issue will be considered further in the “Discussion” section. Cobinamide was not measurable in the plasma of mice that had not received the drug. It is not possible from these data to determine the oral bioavailability of cobinamide, but formal pharmacokinetic studies are currently underway.

Over the 3-month treatment period, the nondiabetic mice gained an average of 4.1 ± 0.8 g, while the diabetic mice lost an average of 0.3 g, with wide variation in weight change among the latter mice (Fig. S4B). Cobinamide was well tolerated by both the nondiabetic and diabetic mice, and did not significantly affect weight in either group, although the cobinamide-treated diabetic mice gained an average of 0.63 g, with wide variation among individual mice (Fig. S4B). Two days prior to euthanasia, a fasting blood glucose and glucose tolerance test showed that the cobinamide-treated mice exhibited the same degree of glucose intolerance as mice that did not receive cobinamide (Fig. S4C and D). Cobinamide had no effect on liver function and renal function tests in the mice (Table SII); due to lack of sufficient blood volume, we were unable to perform complete blood counts, but cobinamide did not affect hematological parameters in mice that received the same amount of cobinamide for 6 months (46).

In the hearts of the diabetic mice, we found increased lipid and protein oxidation as evidenced by increased 8-isoprostane content and protein carbonylation and nitrotyrosine staining, respectively: although some variability existed among the individual diabetic mice, overall they showed a 2.1-, 2.6-, and 3.2-fold increase over the nondiabetic mice for 8-isoprostane (Fig. 4B), protein carbonylation (Fig. 4C and D), and nitrotyrosine, respectively (Fig. 4E and F). Previous workers have found increased 8-isoprostane and ONOO^−^ in hearts of diabetic mice (44, 47); the ONOO^−^ -derived nitrogen dioxide radical can cause nitration of protein tyrosine residues (37). We also found evidence for DNA damage in the hearts of the diabetic mice, as evidenced by increased 8-OH-deoxyguanosine staining (Fig. 4G and H) and decreased DNA integrity using a long-amplicon polymerase chain reaction-based assay (Fig. I) (48, 49). Although the latter assay is not specific for DNA oxidation, the method is quantitative and highly sensitive, and is well-suited for tissue samples. Cobinamide treatment of the diabetic mice significantly reduced all markers of oxidative stress and DNA damage to values similar to those found in the nondiabetic mice (Fig. 4B to I). In all of the above assays, we found no correlation between the plasma cobinamide concentration and the degree of efficacy in individual mice, but this could have been due to relatively small numbers.

Oxidative stress in diabetes contributes to cardiac fibrosis (44, 45), and we found abnormal collagen deposition in the hearts of the diabetic mice (Fig. 4J and K). As observed by others, some of the collagen was present in perivascular regions [Fig. 4J; (50)]. Cobinamide reduced collagen content in the hearts of the diabetic mice essentially to that of nondiabetic mice (Fig. 4J and K).

### Cobinamide does not increase mouse blood pressure at the doses used

We have shown previously that nitric oxide (·NO) reduces Cbi(III) to Cbi(II), and that Cbi(II) binds ·NO with high affinity [(51) and Table 1]. Hence, each cobinamide molecule can neutralize two ·NO molecules, and we have shown that cobinamide can scavenge excess ·NO, both in vitro and in vivo (52, 53). It seemed possible, therefore, that the cobinamide we administered to the mice could have increased their blood pressure, since ·NO is a potent vasodilator. Because diabetes can increase blood pressure, we performed these studies on a parallel set of nondiabetic mice of the same strain and sex and using the same concentration of histidyl-cobinamide in the drinking water as in the above-described studies. We found no difference in the blood pressure of the mice that received cobinamide compared to those that did not receive cobinamide (Fig. S4E).

## Discussion

We found that cobinamide serves as both a SOD and catalase mimetic and that Cbi(II) reacts rapidly with ONOO^−^. These in-vitro data translated well to in-vivo conditions because we found cobinamide is a potent antioxidant in mammalian cells and fruit flies, and reduced oxidative stress and DNA damage in a mouse model of diabetic cardiomyopathy.

Many agents have been shown to have antioxidant properties, but some such as α-tocopherol (vitamin E) and resveratrol have low water solubility and others such as N-acetylcysteine do not predominantly neutralize free radicals. We wanted to compare cobinamide to similar agents, and chose cobalamin, imisopasem, and MnTBAP for the following reasons. Cobalamin is structurally similar to cobinamide, cobalamin(II) reacts readily with O_2_·^−^ and ONOO^−^, both cobalamin(II) and cobalamin(III) react with hydrogen peroxide, and cobalamin has antioxidant effects in several in-vivo systems [Table 1; (13, 14, 17, 19–21)]. Imisopasem and MnTBAP are SOD mimetics, with imisopasem having a relatively high reaction rate with O_2_·^−^, and MnTBAP additionally reacts with ONOO^−^; both agents have been used extensively in cells and animals (24–32) (Table 1).

While some variability existed among the three comparator drugs in terms of efficacy—for example, cobalamin was the only one that improved cell growth in paraquat-treated COS-7 cells and MnTBAP was the only one that enhanced fly survival during paraquat exposure—cobinamide was superior to the three agents in all cell and fly experiments. This may be due to cobinamide’s generally higher reaction rates with O_2_·^−^, H_2_O_2_, and ONOO^−^ compared to the other agents (Table 1). Cobinamide’s favorable reaction with O_2_·^−^ is likely due to its reduction potential of +270 mV, which is about half-way between the one-electron reduction potential of oxygen (−160 mV) and superoxide (+890 mV), and similar to that of the SOD enzymes (∼+300 mV) (54). It is also possible that cobinamide enters cells more readily than the other drugs; this could explain why cobinamide effectively neutralized H_2_O_2_ activation of JNK in H9c2 cells, while cobalamin, which has a reportedly high reaction rate with H_2_O_2_, had no effect. Either way, cobinamide outperformed the comparator drugs, suggesting it may also be effective in the cell and animal systems of oxidative stress where the other drugs have shown efficacy. We used cells and flies in these studies because to have done comparator studies in mice would have required an inordinately large number of animals. Flies allowed us to compare the drugs in a whole animal, and moreover, they are easy to handle and are used in drug development (55).

We found that Cbi(II) reacted faster with hydrogen peroxide than Cbi(III) (Table 1). Consistent with these in-vitro data, Cbi(II) was more effective than Cbi(III) at reducing H_2_O_2_-induced JNK phosphorylation. Cobinamide may exist mainly as Cbi(II) in vivo, because Cbi(III) was reduced quickly to Cbi(II) by ascorbate, cysteine, and GSH. Serum concentrations of ascorbate and cysteine are ∼51 µM and 238 µM, respectively, and the intracellular concentration of GSH is 1 to 10 mM, depending on the cell type (56–58). All of these values are considerably higher than the 0.1 to 0.5 µM concentration, we found in the plasma of mice that had received cobinamide in their drinking water. Moreover, cobalamin is converted intracellularly to its coenzyme forms methyl-cobalamin and adenosyl-cobalamin via enzymatic reduction of cobalamin(III) to cobalamin(II) (59); Cbi(III) could possibly be reduced by this enzyme system.

Superoxide production by mitochondria occurs mostly in the matrix (60). Cobinamide’s nearly complete elimination of MitoSOX Red-induced fluorescence in rotenone- and antimycin A-treated cells suggests cobinamide can enter mitochondria. Similarly, cobinamide’s rapid recovery of oxygen consumption in cyanide- and hydrogen sulfide-poisoned cells and animals is also consistent with mitochondrial uptake (9–11). In addition to reducing intramitochondrial O_2_·^−^, cobinamide recovered the mitochondrial membrane potential. This was likely because O_2_·^−^ activates mitochondrial uncoupling proteins, thereby regulating the membrane potential, and elimination of the O_2_·^−^ allows return of the membrane potential (39).

Although cobinamide reacts with ·NO (Table 1) and ·NO is a major regulator of blood pressure, we found no change in blood pressure of mice treated with cobinamide for several months. Of course, this does not rule out that cobinamide reacted directly with ·NO and thereby could reduce the ·NO concentration, because cobinamide could have simultaneously increased ·NO by neutralizing O_2_·^−^; O_2_·^−^ reacts with ·NO to generate ONOO^−^ at a diffusion-limited rate that exceeds the reaction rate of SOD (54). ONOO^−^ has been shown to be increased in the hearts of diabetic mice (43), and increased ONOO^−^ was a likely cause of the increased tyrosine nitration in the diabetic mice, which was reduced by cobinamide. Although cobinamide can potentially have multiple effects, it reduced nitrosative stress without impairing a physiological function of ·NO.

In a mouse model of human familial aortic aneurysm secondary to a gain-of-function mutation in cGMP-dependent protein kinase, we observed increased oxidative stress in the aortas and aortic media degeneration; administering 1 mM cobinamide in the drinking water, i.e. the same concentration used in the present studies, blocked the increase in oxidative stress and pathological changes, and prevented aneurysm formation (46). The mice showed no clinical or laboratory evidence of toxicity (46). When combined with the current data showing that cobinamide reduced markers of oxidative stress and DNA damage in the hearts of diabetic mice, cobinamide has eliminated oxidation-induced changes in tissues of mice in two separate models at a safe, well-tolerated dose. Cobinamide did not act via improved glucose handling in the current study, because the cobinamide-treated diabetic mice showed the same degree of glucose intolerance as nontreated animals.

We should note several limitations to these studies. First, we used the hypoxanthine–xanthine oxidase-cytochrome c system as the main method to measure cobinamide’s reaction with O_2_·^−^. This system can be impacted by concomitantly generated hydrogen peroxide, but we included catalase in the experiments and showed that it eliminated the generated H_2_O_2_. Moreover, we also measured cobinamide’s reaction with O_2_·^−^ using the spin trap DMPO, and we found similar results as in the cytochrome c system. Second, when we assessed JNK phosphorylation in H9c2 cells, we added H_2_O_2_ to the cells to yield a robust and measurable response. It is possible cobinamide reacted with the H_2_O_2_ prior to entering the cells, but cobinamide would likely also react with intracellular H_2_O_2_ because it reacted with endogenously-produced O_2_·^−^ in cells treated with rotenone and antimycin A. Furthermore, cobalamin(III), with a reportedly high reaction rate with H_2_O_2_ was without effect, suggesting that neutralization of the H_2_O_2_ did not occur outside the cell. Third, we used male mice only in the studies of diabetes-induced oxidative stress. Female mice might respond differently to cobinamide, but cobinamide was equally effective in male and female mice with the gain-of-function mutation in cGMP-dependent protein kinase (46); moreover, in the fly experiments, we used males and females, and they responded similarly to cobinamide’s antioxidant effects. Fourth, although we tried to compensate for the increased water consumption of the diabetic mice by decreasing the cobinamide concentration in their drinking water, this was not strictly possible because the mice were housed three to four per cage (for socialization reasons, our IACUC discourages single housing of mice). Variation in water consumption likely led to the wide variation in plasma cobinamide concentrations among the diabetic mice. Finally, we did not assess cardiac function in the mice, because the study was not powered for echocardiographic measurements. Future studies will include such assessments.

In addition to diabetes, increased oxidative stress occurs in a wide variety of other diseases, for example, cardiovascular disorders such as heart failure and ischemia-reperfusion injury, neurodegenerative diseases such as Alzheimer’s disease and multiple sclerosis, and acute and chronic inflammatory conditions such as bacterial or viral infections and rheumatoid arthritis (61–63). In several of these diseases, clinical trials of antioxidants have yielded mixed results, possibly due to inadequate drug efficacy (62, 63). A need exists for more effective antioxidants (64). Compared to most other antioxidants, cobinamide is unique because it serves as both a superoxide and catalase mimetic, it can scavenge excess nitric oxide, and it reacts with peroxynitrite (Table 1). Cobinamide’s versatility and potency in neutralizing reactive oxygen and nitrogen species may explain why it was superior to three other antioxidants in the present studies, and suggest it has potential utility in treating diseases characterized by increased oxidative stress.

## Methods

### Additional methods are in the Supplementary Materials

#### Cobinamide synthesis and nomenclature

Cobinamide was synthesized from cobalamin by base hydrolysis using freshly made cerium hydroxide (from cerium chloride), and purified over two reversed phase resin columns as described previously (4). The resulting product was >98% pure as determined by high-performance liquid chromatography (HPLC) with UV detection and by mass spectrometry. Under ambient conditions, the cobalt is in the +3 oxidation state, and in aqueous solutions at neutral pH, a water and hydroxyl group are coordinated to the cobalt, i.e. it is aquohydroxo-cobinamide (Fig. 1A). Throughout the text, this species is referred to as “cobinamide,” but when it is important to delineate its oxidation state, it is referred to as Cbi(III). To generate Cbi(II), we added two molar equivalents of either ascorbic acid or sodium borohydride to Cbi(III). The ascorbic acid was removed by passing the solution over an anion exchange column (Dowex 1) and the borohydride was decomposed in dilute acid. The resulting Cbi(II) remains in the reduced state for several hours, even on exposure to air. In the mouse experiments, we administered cobinamide as histidyl-cobinamide (cobinamide with two bound histidine molecules) because it is stable in aqueous solutions, allowing it to be used in drinking water. It was generated by adding three molar equivalents of L-histidine to cobinamide.

#### Measurement of cobinamide reaction with superoxide

We studied the reaction of cobinamide with O_2_·^−^ in two different systems, generating O_2_·^−^ using a hypoxanthine–xanthine oxidase system.

The first system contained 100 µM hypoxanthine and 0.1 unit xanthine oxidase in 20 mM sodium phosphate buffer, pH 7.1. The amount of O_2_·^−^ was measured by following reduction of ferric-cytochrome c (70 µM) at 550 nm as described previously (65). Catalase was included (32 units per sample), because xanthine oxidase generates both O_2_·^−^ and H_2_O_2_, and the latter can reoxidize the ferro-cytochrome c product (34, 66). The reaction was followed for 10 min. A no substrate blank lacking hypoxanthine was included.

The second system contained 1 mM hypoxanthine, 0.04 U xanthine oxidase, and 0.26 M DMPO in phosphate-buffered saline, pH 7.4 (PBS). Samples were transferred to a capillary tube, and introduced into the EPR cavity of a Magnettech MiniScope MS5000. DMPO-OH signals arising from the DMPO-OOH spin adduct were measured at 37°C for 5 min. The area under the DMPO-OOH peak was calculated using Origin 2022b software. To avoid variability across runs, signal amplitudes were normalized to the intensity of simultaneously recorded Mn reference signals originating from ZnS: Mn^2+^ fixed within the EPR cavity.

In both systems, the experiments were conducted in the absence and presence of the indicated concentrations of Cbi(III) or Cbi(II) (65).

#### Assessment of mitochondrial superoxide content

H9c2 cells were plated on glass cover slips in 24 well dishes, and 16 h later the medium was changed to phenol red-free DMEM supplemented with 20 mM Hepes, 0.1% fetal bovine serum, and 0.5% bovine serum albumin. The cells were incubated for 30 min at room temperature with 5 µM rotenone or 10 µM antimycin A in the absence or presence of 2.5 µM of the indicated antioxidant, with 5 µM MitoSOX Red added during the last 10 min. MitoSOX Red is a dihydroethidium derivative containing triphenyl-phosphonium, which localizes it to respiring mitochondria. Its reaction with O_2_·^−^ yields 2-hydroxy-mitoethidium (2-OH-Mito-E^+^), whereas its reaction with other oxidative species yields mitoethidium (Mito-E^+^) (67). These two oxidative derivatives have overlapping fluorescence spectra, but they can be distinguished by HPLC (68). We showed that rotenone and antimycin A increased 2-OH-Mito-E^+^ about two-fold, without increasing Mito-E^+^ (Table S1). Thus, any increased fluorescence observed on treating cells with rotenone or antimycin could be ascribed to an increase in 2-OH-Mito-E^+^, and decreased fluorescence by an antioxidant in rotenone- or antimycin A-treated cells was likely from a decrease in 2-OH-Mito-E^+^.

#### Assessment of mitochondrial membrane potential

For assessment of the mitochondrial membrane potential, H9c2 cells were pre-incubated with 10 µM JC-1 for 10 min at room temperature; JC-1 is a cationic dye that exhibits potential-dependent accumulation in mitochondria indicated by a fluorescence emission shift from green to red (40). The cells were then washed once with PBS, placed back in the DMEM experimental medium, and incubated with the indicated drugs for 30 min. At the end of the incubation period, nuclei of both the MitoSOX Red- and JC-1-treated cells were stained with 2 mg/ml 4′,6-diamidino-2-phenyl-indole (DAPI) for 3 min, washed once with PBS, and returned to the DMEM experimental medium. The cover slips were removed from the wells, mounted in the DMEM experimental medium, and red (MitoSOX Red and JC-1 aggregates), green (JC-1 monomers), and blue (DAPI) fluorescence intensity was assessed using a Keyence BZ-X700 fluorescence microscope at the following paired excitation/emission wavelengths, respectively: 560/630, 490/525, and 360/460 nm. We showed in control experiments that cobinamide did not interfere with the fluorescence signal by adding cobinamide to the cells immediately before observing fluorescence.

#### Generation and treatment of diabetic mice

Male C57BL/6NHsd mice (20 weeks old) were housed three to four animals per cage in a temperature-controlled environment with a 12-h light/dark cycle and fed standard rodent chow with ad-libitum access to food and water. After 1 week of acclimatization, mice weighing 30 ± 3 g were injected intraperitoneally for five consecutive days with either saline (*n* = 20) or 50 mg/kg streptozotocin (*n* = 20) (69). Thirteen days after the last streptozotocin injection, the blood glucose concentration was measured after a 6 h fast using a commercial glucometer; 16 of the 20 streptozotocin-injected mice had a blood glucose concentration >270 mg/mL and were considered diabetic. The 16 diabetic mice and a corresponding number of the saline-injected, nondiabetic mice were split randomly in half to receive either plain drinking water or histidyl-cobinamide in the drinking water. The four groups of animals are referred to as: diabetic vehicle (D-Veh); diabetic, cobinamide-treated (D-Cbi); nondiabetic, vehicle (ND-Veh); and nondiabetic, cobinamide-treated (ND-Cbi). The histidyl-cobinamide concentration was 1 mM for the ND-Cbi mice, but because the diabetic mice drank more than the nondiabetic mice, the cobinamide concentration for the D-Cbi mice was decreased accordingly. The water was replenished twice a week.

After 3 months of treatment, the mice were weighed, fasted for 6 h, and then underwent an intraperitoneal glucose tolerance test (IPGTT) using 2 g/kg of a 20% glucose solution. Blood glucose concentrations were measured just before the glucose injection (time 0, fasting specimen) and at 15, 30, 60, and 120 min following the injection. One mouse in the D-Veh group and two mice in the D-Cbi group were found not to be diabetic, with a normal fasting blood glucose concentration and normal IPGTT; they were excluded from further study. Two days later, the mice were euthanized by inducing deep anesthesia with 200 mg/kg ketamine and 40 mg/kg xylazine administered by intraperitoneal injection, followed by exsanguination via an open cardiac puncture. The hearts were removed quickly and dipped into ice-cold PBS to remove excess blood; the apex was cut off and fixed in 4% formaldehyde, with the remainder of the heart flash frozen in liquid nitrogen. Paraffin-embedded blocks were made from the formaldehyde-fixed apical samples and cut into 5 µM thick sections that were mounted on glass slides.

#### Assessment of lipid 8-isoprostane, protein nitrosylation and carbonylation, DNA damage, and collagen deposition in heart samples

8-isoprostane in heart extracts was measured by ELISA. Frozen heart pieces (10 to 20 mg) were pulverized in liquid nitrogen, and then processed according to the manufacturer’s recommendation. Standard curves were included in each assay over a range bracketing sample values. Each sample was measured in duplicate at two different dilutions. The data are expressed as picogram per milligram of wet tissue.

8-OH-deoxyguanosine and nitrotyrosine were assessed by immunohistochemistry. Slides with mounted cardiac sections were incubated for 10 min in 10 mM sodium citrate buffer, pH 6.0 at 80 to 85°C. Endogenous peroxidase activity was quenched in 3% H_2_O_2_ for 10 min. After blocking with 2% normal goat serum, slides were incubated overnight at 4°C with an anti-8-OH-deoxyguanosine or antinitrotyrosine primary antibody, followed by a horseradish peroxidase-conjugated secondary antibody. After development with 3-diaminobenzidine (Vector Laboratories) (46), nuclei were counterstained with hematoxylin, and images were analyzed with a Hamamatsu Nanozoomer Slide scanning system. The number of brown-stained cells were counted from five separate areas of 100 cells per area.

Protein carbonylation was assessed using the Oxyblot protein oxidation system from EMD Millipore. Frozen tissue was pulverized in liquid nitrogen and extracted in RIPA buffer containing 50 mM dithiothreitol and protease inhibitors, with half of the extract (∼5 µg protein) incubated with 2,4-dinitrophenylhydrazine (DNP). The samples were subjected to PAGE-immunoblotting, with carbonylated proteins detected using an anti-DNP antibody.

DNA damage was measured in an 8.7 kb fragment of the mouse β-globin gene using a long-amplicon, quantitative polymerase chain reaction-based assay (48, 49). Briefly, 10 to 20 mg of frozen heart tissue was pulverized on dry ice, and large genomic DNA was purified using Qiagen Genomic-tip 20/G columns as recommended by the manufacturer. The DNA was quantified using a nanodrop spectrophotometer, diluted to 6 μg/μL and then quantified again using Quant-iT PicoGreen dsDNA Assay Kit (Invitrogen). The DNA was diluted to 1 ng/μL, and 5 and 10 ng were added to a polymerase chain reaction containing 20 pmol each of 5’ and 3’ primers, and LongAmp Hot Start Taq Master Mix (New England Biolabs, Inc.). Cycling conditions consisted of 94°C for 2 min, followed by 27 cycles of 94°C for 15 s and 63°C for 12 min. The PCR product was subjected to electrophoresis on a 0.5% agarose gel containing ethidium bromide and was quantified using a LiCor imaging system. Any DNA damage including modified bases and strand breaks halt synthesis such that the amount of generated DNA is inversely proportional to the amount of damage.

Collagen deposition was assessed by staining slides containing mounted cardiac sections with Mallory’s trichrome stain, which stains collagen fibers blue. The % blue area in a total heart section was measured using Image-Pro Premier software (Version 9.0, Media Cybernetics).

### Animal experiments

All mouse experiments were conducted according to the National Academies of Sciences, Engineering, and Medicine Institute for Laboratory Animal Research Guide to the Care and Use of Laboratory Animals and were approved by the Institutional Animal Care and Use Committee (IACUC) at the University of California, San Diego. IACUC approval is not required for work with fruit flies.

### Statistical analysis

Statistical tests were conducted using GraphPad Prism Statistics Software Version 7.04. Data in bar graphs are presented as the mean ± SD. The data for the cell-based assays in Fig. 3 and Fig. S3 were analyzed by one-way ANOVA with *P* < 0.01 in all cases; paired comparisons of all conditions were made using Tukey’s multiple comparisons test. The data from mouse experiments in Fig. 4 and Fig. S4 were analyzed by two-way ANOVA with significant interaction (*P* < 0.05) in all cases; paired comparisons were made using Sidak’s multiple comparisons test. A *P*-value < 0.05 was considered significant.

## Supplementary Material

pgac191_Supplemental_Files

## Data Availability

All data are contained within the manuscript.
