# Supplementary material for: Cobinamide is a strong and versatile antioxidant that overcomes oxidative stress in cells, flies, and diabetic mice
Source: PNAS Nexus. 2022 Sep 14;1(4):pgac191. doi: 10.1093/pnasnexus/pgac191 (PMC9578022; doi:10.1093/pnasnexus/pgac191)
Supplement: pgac191_Supplemental_Files [file pgac191_supplemental_files.zip › PNASNEXUS-PNASNEXUS-2022-00476-s01.docx]

Supplementary Material

Cobinamide is a strong and versatile antioxidant that overcomes oxidative stress in cells, flies, and diabetic mice

Stephen Chang, John Tat, Shyamsundar Pal China, Hema Kalyanaraman, Shunhui Zhuang, Adriano Chan, Cassandra Lai, Zoran Radic, Darren E. Casteel, Engy A. Abdel-Rahman, Renate B. Pilz, Sameh S. Ali, and Gerry R. Boss

Content:

Additional Methods

Figure S1 Cbi(III) and Cobalamin(III) Reaction with Superoxide

Figure S2 Reaction Rates of Cbi(III) and Cbi(II) with H_2_O_2_; Absorbance Spectra after Adding H_2_O_2_ to Cbi(III) and Cbi(II); H_2_O_2_ Reaction Tracing

Figure S3 Assessment of Mitochondrial O_2_·^-^ and Mitochondrial Membrane Potential in Antimycin A-treated H9c2 Cells; Cobinamide Spectrum in the Presence of Paraquat, Rotenone, and Antimycin A; JNK Phosphorylation in H9c2 Cells

Figure S4 Plasma Cobinamide Concentration, Weight, Blood Glucose, and Glucose Tolerance Test in Diabetic and Non-diabetic Mice; Blood Pressure in Parallel Group of Non-diabetic Mice

Table S1 2-Hydroxymitoethidium (2-OH-Mito-E^+^) and Mitoethidium (Mito-E^+^) in MitoSOX-treated Cells

Table S2 Clinical Chemistry Panel of Mice

Table S3 Antibody Source and Use

Video: Cbi(II) Reaction with Superoxide (uploaded as separate file)

References

**ADDITIONAL METHODS**

**Materials**

Hydroxo-cobalamin (referred to as “cobalamin”), L-histidine, hypoxanthine, MnTBAP, paraquat, 5,5-dimethyl-1-pyrroline N-oxide (DMPO), bovine milk xanthine oxidase, bovine liver catalase, and horse heart cytochrome c were from Sigma-Aldrich. Hydrogen peroxide and anhydrous DMSO were from Fisher Scientific, imisopasem manganese (imisopasem) was from MedChemExpress, peroxynitrite and an 8-isoprostane (8-*iso*-prostaglandin F_2α_) ELISA system were from Cayman Chemical, and MitoSOX Red and JC-1 were from Invitrogen. A H_2_O_2_-specific probe and Free Radical Analyzer (TBR4100) were from WPI Instruments. Source of antibodies is in Table S3.

**Measurement of UV-Visible Absorption Spectra**

The UV-visible absorption spectra of Cbi(III) and Cbi(II), in the absence and presence of other compounds, were recorded on a Kontron 860 dual-beam spectrophotometer at a scanning rate of 300 nm/min. Spectra were recorded at room temperature using 1 cm path length cuvettes. In some experiments, the cobinamide was dissolved in DMSO.

**Measurement of Cobinamide Reaction with Hydrogen Peroxide**

Hydrogen peroxide was added to PBS resulting in an immediate and large increase in current as measured by an H_2_O_2_-specific electrochemical probe. Over the 20 min time frame of the experiment, a slight decrement in signal occurred, possibly due to the presence of transition metals in the PBS (Figure S2G, inset). In the presence of Cbi(III) or Cbi(II), a linear decrease in current occurred over time proportionate to the Cbi(III)/Cbi(II) concentration (Figure S2G, main figure, and Fig. 2A,C). Amperage was converted to H_2_O_2_ concentration using standard curves. Experiments were conducted at room temperature with constant stirring of the reaction solution.

**Measurement of Cobinamide Reaction with Peroxynitrite**

Varying concentrations of Cbi(II) from 1 to 10 µM were mixed with 10 µM ONOO^-^ in a stopped-flow instrument, and oxidation of Cbi(II) to Cbi(III) was measured by following the decrease in UV absorption at 315 nm. The Cbi(II) was in sodium phosphate, pH 7.4, and due to the instability of ONOO^-^ at neutral pH, it was in 10 mM NaOH; on mixing, the pH of the combined solutions rose to 7.8. Experiments were conducted at room temperature using an Applied Photophysics SX.18 MV instrument equipped with a diffraction grating symmetrical Czerny-Turner monochromator at an optical path length of 2 mm. Absorption readings were taken every 1.25 msec for 0.5 sec and plotted versus time. A biphasic linear regression analysis (Prism 7.04 software) yielded a dominant, concentration-dependent k observed, which was plotted versus the Cbi(II) concentration to determine the reaction rate constant.

**Cell Culture**

H9c2 rat embryonal cardiomyocytes [H9c2(2-1), CRL-1446] and COS-7 African green monkey fibroblasts (CRL-1651) were purchased from the American Type Culture Collection. They were cultured in Dulbecco’s Modified Eagle’s Medium (DMEM) supplemented with 10% fetal bovine serum. The H9c2 cells expressed ventricular myosin light chain 2 (vMLC2) and cardiac troponin I (cTnI), and both cell lines were Mycoplasma negative. For experiments, logarithmically-growing cells were seeded the day before as indicated.

**Measurement of Cell Growth**

COS-7 cells were seeded at 50,000 cells per well in a six-well plate. The next day they were exposed to 1 mM paraquat for 3 h in the absence or presence of 100 µM of the indicated drugs. After the drugs were removed, the cells were washed twice with PBS and incubated in drug-free medium for 48 h, at which time they were counted using a hemocytometer.

**Assessment of JNK Phosphorylation**

H9c2 cells were incubated for 30 min in the absence or presence of 100 µM H_2_O_2_, without or with 100 µM of the indicated drugs. The cells were extracted *in situ* in a sodium dodecyl sulfate-based buffer, and the extracts were heated. Proteins were separated by polyacrylamide gel electrophoresis, and phospho-JNK(Thr^183^/Tyr^185^), total JNK, and GAPDH were assessed by immunoblotting. Band intensity was determined by densitometric scanning using a Li-Cor Odyssey instrument over a range where the band intensity was linear.

**Exposure of Flies to Oxidative Stress and Rescue by Antioxidants**

Paraquat and the indicated drugs were added to fly food by melting the food with gentle heating, and mixing in concentrated solutions of paraquat and the drugs to achieve final concentrations of 20 mM paraquat and 0.8 mM drug. Oregon-R *Drosophila melanogaster* of both sexes and variable age were placed into vials containing the treated food, and the number of live flies was recorded over a seven-day period. Each vial contained ten flies and the experiment was repeated seven times for a total of 70 flies per condition.

**HPLC Analysis of MitoSOX Oxidation Products**

H9c2 cells were grown to sub-confluence in 6 well culture dishes. The medium was changed, and the cells were incubated for 30 min with 10 µM rotenone, 10 µM antimycin A, or 2.5 µM cobinamide. During the last 10 min of the incubation, 15 µM MitoSox Red was added. The cells were washed once with ice-cold PBS, and harvested by scraping with a rubber policeman. The cells were then processed according to Zielonka et al (1). Briefly, the cells were centrifuged and the pellet was extracted in 0.1% Triton X-100. Proteins were precipitated from the extract using 0.2 M HClO_4_ in methanol, KPO_4_, pH 2.6 was added, and the resulting KClO_4_ precipitate was removed by centrifugation. The supernatant was applied to a C18 reversed-phase resin column eluted with a gradient from 0.1% trifluoroacetic acid in water to 0.1 % trifluoroacetic acid in acetonitrile using a Model 7000 Hitachi HPLC system. The eluate was monitored by UV absorption at 480 nm, and 2-OH-Mito-E^+^ and mitoethidium were identified by comparing their elution times to standards generated as described by Zielonka et al (1). Areas under the peaks were integrated per the system software.

**Measurement of Plasma Cobinamide Concentration**

Whole blood from the mice was anticoagulated with 5 mM ethylenediamminetetraacetic acid and centrifuged at 5,000 X g to remove cells. The resulting plasma was extracted and cobinamide was measured by high performance liquid chromatography coupled with mass spectrometry as described [Analytical and Diagnostic Solutions, LLC (2)].

**Measurement of Mouse Blood Pressure**

The systolic and diastolic blood pressures of mice were measured on the tail artery using the CODA Non-invasive Blood Pressure System (Kent Scientific) according to the manufacturer’s recommendation. Recorded values are the mean of three consecutive readings.


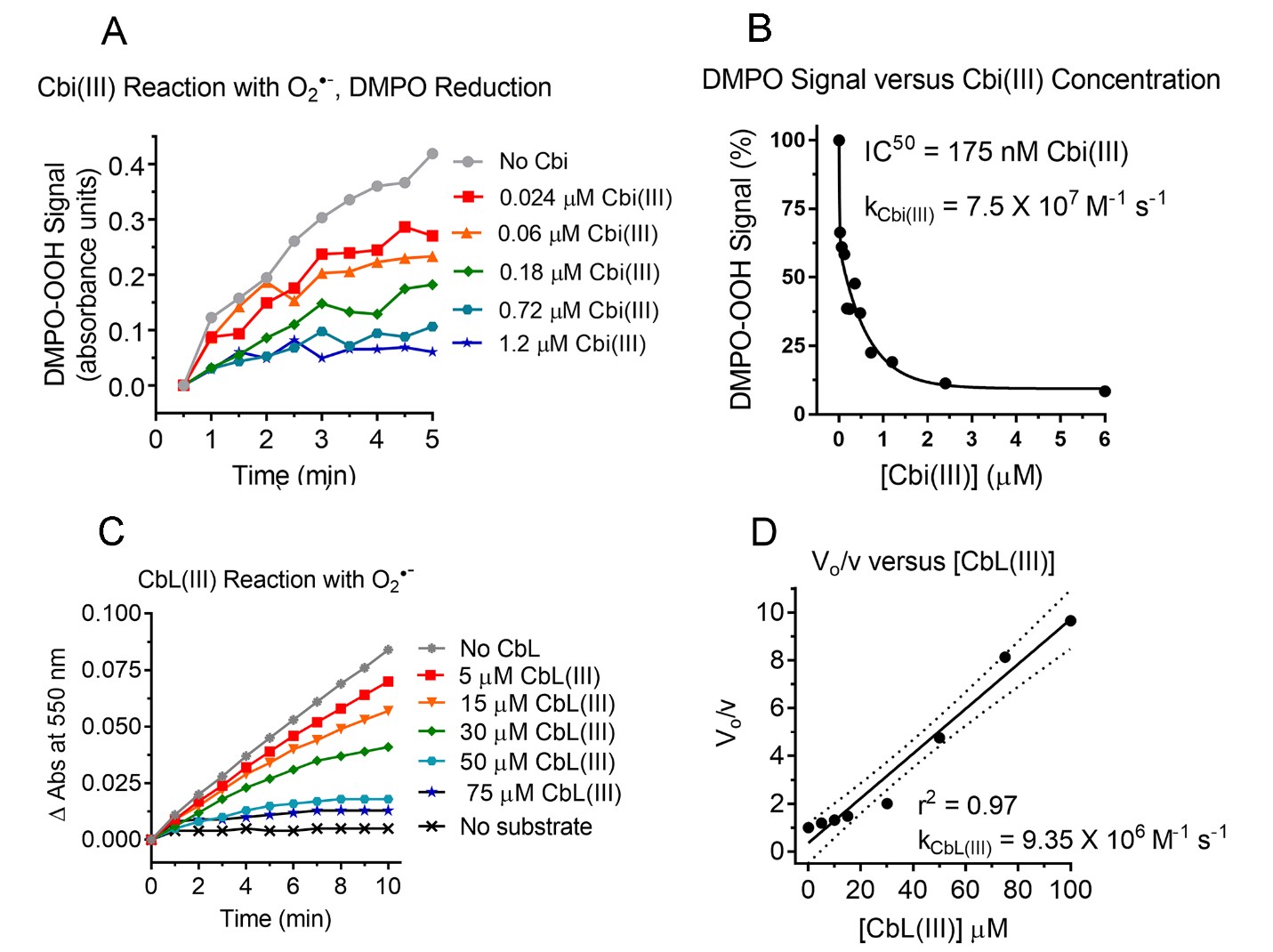


**Figure S1. Cbi(III) and Cobalamin(III) Reaction with Superoxide.**

**A.** Superoxide was generated by the hypoxanthine-xanthine oxidase system. The amount of O_2_·^-^generated was measured using the spin-trap DMPO and the observed DMPO-OH signal arising from DMPO-OOH spin adduct was quantified over 5 min. The effect of Cbi(III) concentrations ranging from 0.024 µM to 6 µM was assessed; for clarity sake, data are shown for only 5 of the 11 Cbi(III) concentrations tested.

**B.** The data from Panel A were plotted as the percent of the DMPO-OOH signal versus the Cbi(III) concentration, yielding an IC_50_ for Cbi(III) of 175 nM. The apparent rate constant for Cbi(III) was calculated according to the following formula: k_Cbi(III)_ = k_DMPO_ X [DMPO]/IC_50_ Cbi(III), where k_DMPO_ = 50 M^-1^ s^-1^ (3) and [DMPO] = 260 mM.

**C****.** Increasing concentrations of cobalamin(III) [CbL(III)] were incubated in a hypoxanthine-xanthine oxidase-cytochrome c-catalase system for measuring O_2_·^-^. The change in absorbance (ΔAbs) at 550 nm is plotted versus time. A no substrate (no hypoxanthine) control was included.

**D.** The data from Panel D were plotted as V_o_/v versus the cobalamin concentration and yielded an apparent rate constant of 9.35 X 10^6^ M^-1^ s^-1^. Dotted lines show 95% confidence intervals.


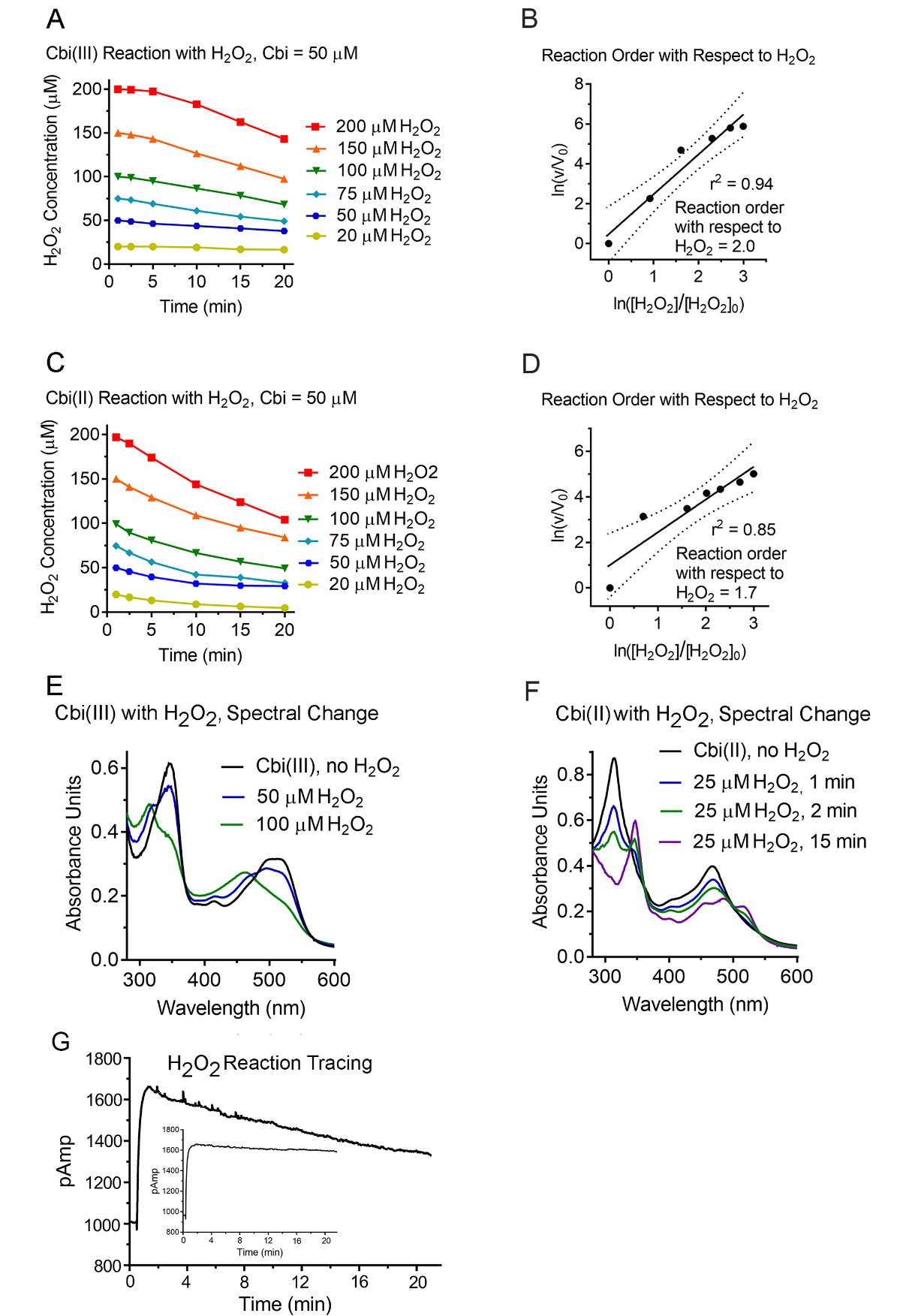
**Figure S2. Reaction Rates of Cbi(III) and Cbi(II) with H_2_O_2_; Absorbance Spectra after Adding H_2_O_2_ to Cbi(III) and Cbi(II); H_2_O_2_ Reaction Tracing**

**A-D.** Increasing concentrations of hydrogen peroxide were added to solutions containing 50 µM Cbi(III) or Cbi(II), and the H_2_O_2_ concentration was measured over time (**A,C**). **B,D**. The ln(v/V_0_) was plotted against ln([H_2_O_2_]/[H_2_O_2_]_0_) yielding reaction orders with respect to H_2_O_2_ of 2 and 1.7 for Cbi(III) (**B**) and Cbi(II) (**D**), respectively. Dotted lines show 95% confidence intervals.

**E.** To a 25 µM solution of Cbi(III) was added H_2_O_2_ to a final concentration of 50 or 100 µM, and the UV-visible spectrum was recorded 10 min later.

**F**. To a 25 µM solution of Cbi(II) was added 25 µM H_2_O_2_, and the UV-visible spectrum was recorded 1, 2, and 15 min later.

**G.** Hydrogen peroxide (final concentration 50 µM) was added to PBS containing 60 µM Cbi(III) and the change in amperage was measured over time using a H_2_O_2_-specific electrode. Inset: Same as in main figure, but no Cbi(III) present.


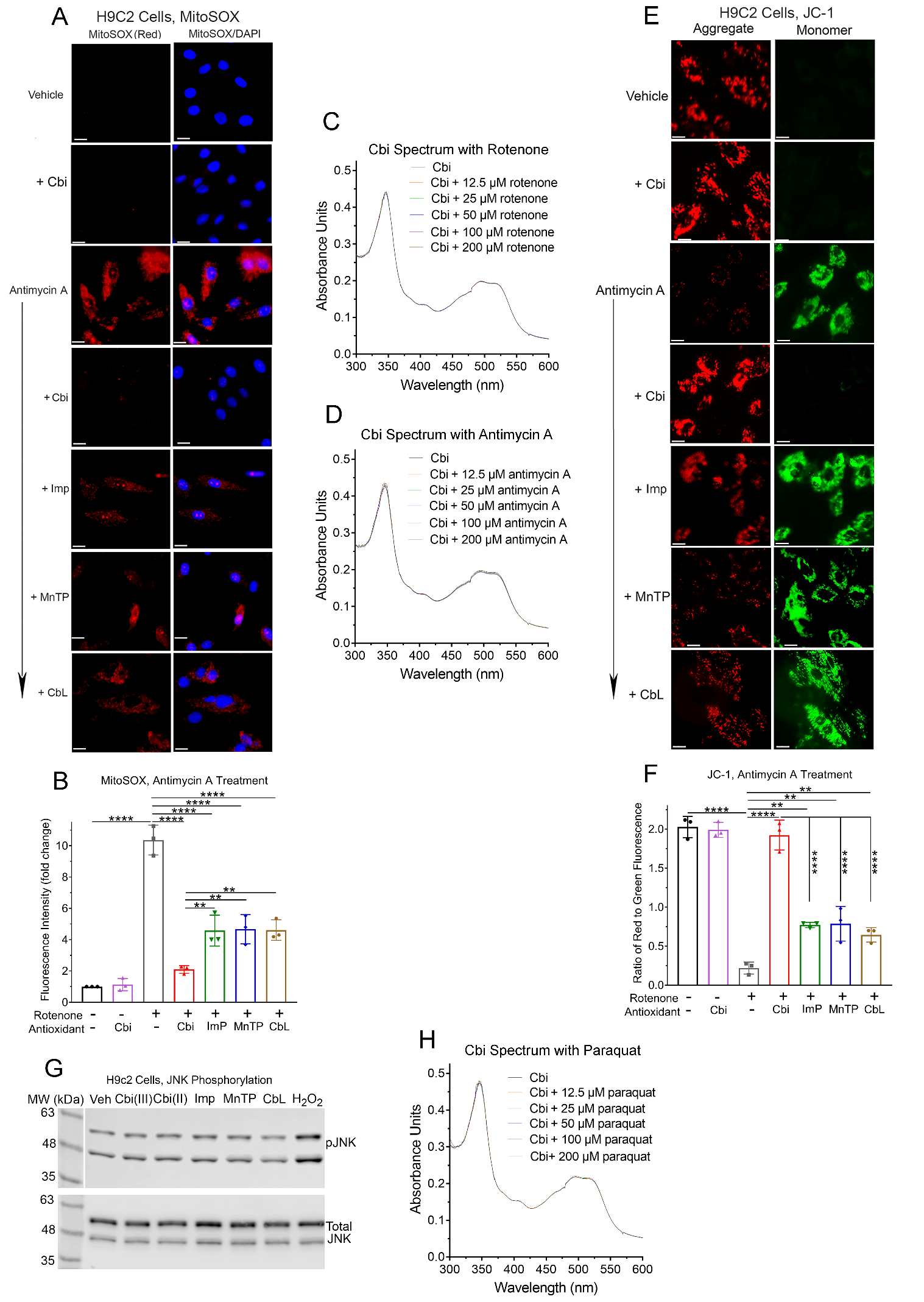
**Figure S3. Assessment of Mitochondrial O_2_·^-^ and Mitochondrial Membrane Potential in Antimycin A-treated H9c2 Cells; Cobinamide Spectrum in the Presence of Rotenone, Antimycin A, and Paraquat; JNK Phosphorylation in H9c2 Cells**

**A,B.** H9c2 cells were incubated for 30 min with vehicle, 2.5 µM cobinamide, or 10 µM antimycin A without or with 2.5 µM cobinamide, imisopasem, MnTBAP, or cobalamin; 5 µM MitoSOX was added during the last 10 min. The cells were analyzed by fluorescent microscopy. **A.** Equal-sized representative areas are shown for MitoSOX staining (red fluorescence) and the same area showing merged MitoSOX and DAPI staining. **B.** The amount of red fluorescence was quantified by Image J analysis. Data are the mean ± SD of three independent experiments; in each experiment two separate equal-sized areas were analyzed containing ~ 75 cells per area.

**C,D.** Increasing concentrations of rotenone (**C**) or antimycin A (**D**) were added to a 25 µM solution of cobinamide, and the UV-visible spectra were recorded from 300 to 600 nm.

**E,F.** H9c2 cells were incubated for 10 min with 10 µM JC-1, washed once with PBS, and then incubated for 30 min with vehicle, 2.5 µM cobinamide, or 10 µM antimycin A without or with the indicated drugs as described in Panels A,B. **E.** Cells were visualized under a fluorescence microscope and equal-sized representative areas are shown for each condition, with red and green fluorescence shown for the same area. **F.** The amount of red and green fluorescence was quantified by Image J analysis and the red to green ratio was calculated. Data are the mean ± SD of three independent experiments; in each experiment two separate equal-sized areas were analyzed containing ~ 75 cells per area.

**G.** H9c2 cells were incubated for 30 min with vehicle (Veh), 100 µM of the indicated drugs without H_2_O_2_, or 100 µM H_2_O_2_ alone. The cells were extracted in a SDS-based buffer, proteins were resolved by PAGE, and phospho-JNK(Thr^183^/Tyr^185^) (pJNK) was identified by immunoblotting (upper blot). A replicate blot was probed with an antibody against total JNK (lower blot). The two isoforms of pJNK have observed molecular weights of 46 and 54 kDa. In the upper blot, the molecular weight marker was shifted over one lane. Similar results were found in two other experiments.

**H.**  Increasing concentrations of paraquat were added to a 25 µM solution of cobinamide, and the UV-visible spectra were recorded from 300 to 600 nm.

For Panels B and F, the slides were analyzed by an operator who was blinded to the specific conditions.

Cbi, cobinamide; Imp, imisopasem; MnTP, MnTBAP; CbL, cobalamin. White scale bar in A and E is 10 µM in length.

The data in Panels B and F were analyzed by a one-way ANOVA (p < 0.0001) followed by Tukey’s multiple comparison test of all conditions; ** and **** indicate p < 0.01 and 0.0001, respectively for the indicated paired comparisons. Comparison of vehicle-treated cells to cells treated with cobinamide with or without antimycin A was not significant, whereas comparison of vehicle-treated cells to antimycin A-exposed cells treated with imisopasem, MnTBAP, or cobalamin was significant, with the p values at least < 0.001.


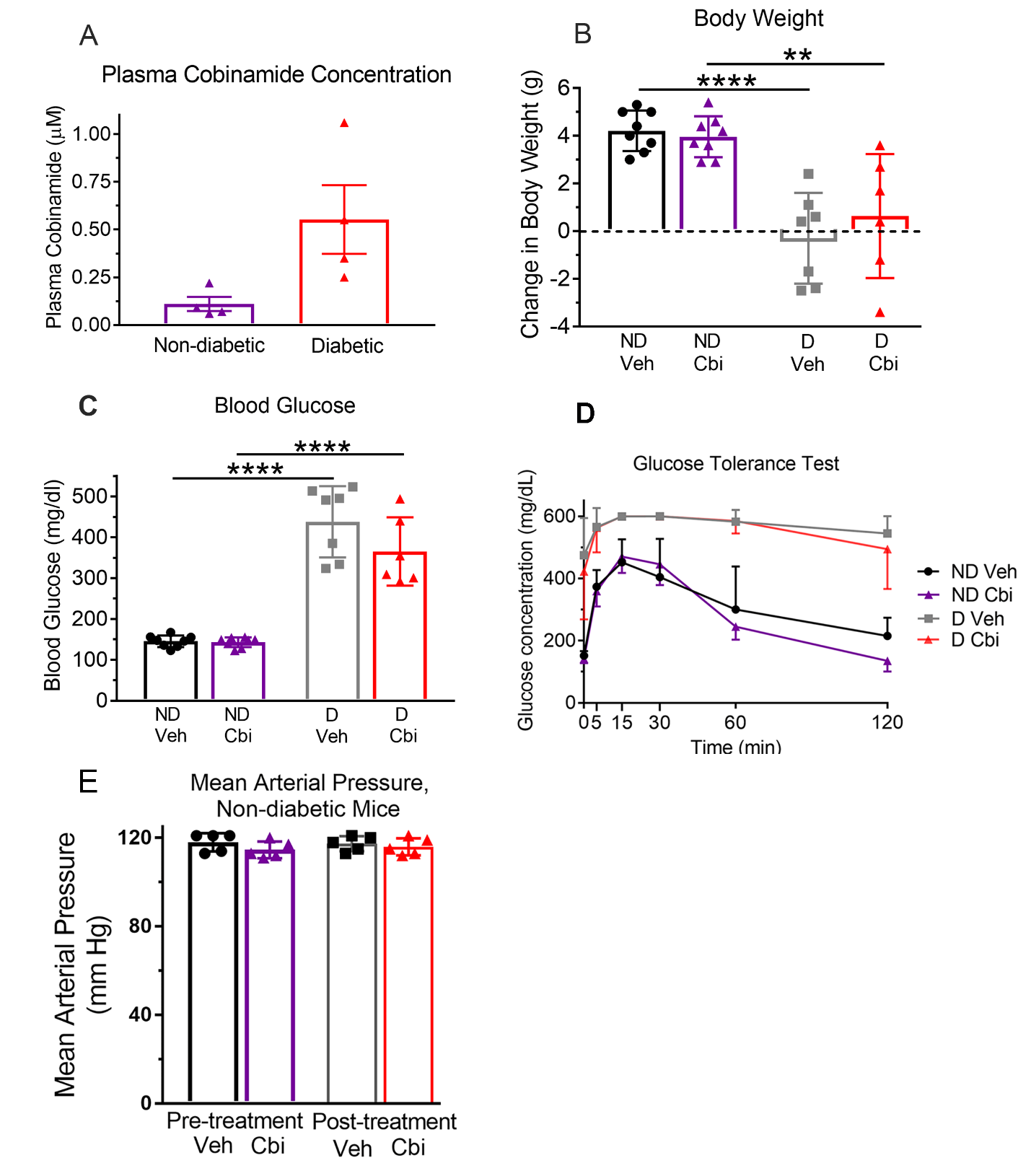


**Figure S4. Plasma Cobinamide Concentration, Weight, Blood Glucose, and Glucose Tolerance Test in Diabetic and Non-diabetic Mice; Blood Pressure in Parallel Group of Non-diabetic Mice**

**A.** Plasma cobinamide concentration was measured by high performance liquid chromatography-mass spectrometry from four non-diabetic and four diabetic mice that had received 1 mM histidyl-cobinamide in the drinking water; the four mice from each group were selected randomly from the full group.

**B.** Mice were weighed at the beginning of the study and one day prior to euthanasia; the change in their body weight over the study period is shown.

**C.** Mice were fasted for 6 h, and blood was obtained from a small nick in the tail two days prior to euthanasia. The blood glucose concentration was measured using a commercial glucometer.

**D.** An intraperitoneal glucose tolerance test was performed on the mice shown in Panel C. The glucometer had a maximal readout of 600 mg/dl, and thus, some of the values may have been higher. For sake of clarity, error bars are shown in one direction only.

**E.** The blood pressure of five non-diabetic mice was measured before (pre-treatment) and after receiving 1 mM histidyl-cobinamide in the drinking water for four months (post-treatment). Five age-matched mice that did not receive cobinamide (Veh) were included. The mean arterial pressure was calculated as the sum of the diastolic blood pressure plus one-third of the difference between the systolic and diastolic blood pressure.

ND, non-diabetic; D, diabetic; Veh, vehicle; Cbi, histidyl-cobinamide.

The data in Panels B and C were analyzed by a two-way ANOVA (interaction, p < 0.05) followed by Sidak’s multiple comparison test; ** and **** indicate p < 0.01 and 0.0001, respectively for the indicated paired comparisons.

Table S1. 2-Hydroxy-mitoethidium (2-OH-Mito-E^+^) and Mitoethidium (Mito-E^+^) in MitoSOX-treated Cells

H9c2 cells were incubated for 30 min with vehicle, 10 µM rotenone, 10 µM antimycin A, or 2.5 µM cobinamide; 15 µM MitoSOX Red was added during the last 10 min. The cells were extracted, and the extracts analyzed by HPLC as described in methods. The numbers represent the integrated areas for the 2-OH-Mito-E^+^ and Mito-E^+^ peaks per million cells. Each value is the mean of two independent experiments.

| Species | Vehicle | Rotenone | Antimycin A | Cobinamide |
| --- | --- | --- | --- | --- |
| 2-OH-Mito-E^+^ | 1977 | 3603 | 4734 | 2180 |
| Mito-E^+^ | 974 | 852 | 996 | 1031 |

Table S2. Clinical Chemistry Panel of Mice.

C57BL/6NHsd male mice were made diabetic with streptozotocin, with half of both the control mice (vehicle-injected) and diabetic mice receiving 1 mM cobinamide in their drinking water for three months. Blood was collected at the time of euthanasia, and plasma was analyzed in a VetScan 2 Analyzer. ND-Veh = Non-diabetic, vehicle; ND-Cbi = Non-diabetic, cobinamide; D-Veh = diabetic, vehicle; D-Cbi = diabetic, cobinamide. The data are the mean ± SD of 4 animals randomly selected from each group. Analysis by two-way ANOVA showed no difference among the four groups for any of the measured parameters.

| Parameter | ND-Veh | ND-Cbi | D-Veh | D-Cbi |
| --- | --- | --- | --- | --- |
| Albumin (g/dl) | 4.1 ± 0.6 | 3.4 ± 0.4 | 3.5 ± 0.7 | 3.5 ± 0.4 |
| Alkaline phosphatase (U/L) | 13 ± 6 | 16 ± 5 | 9.5 ± 5 | 8.8 ± 4 |
| Alanine transaminase (U/L) | 23 ± 5 | 19 ± 9 | 32 ± 11 | 34 ± 22 |
| Total bilirubin (mg/dl) | 0.2 ± 0.1 | 0.2 ± 0.05 | 0.3 ± 0.1 | 0.2 ± 0.06 |
| Blood urea nitrogen (mg/dl) | 23 ± 7 | 19 ± 3 | 30 ± 3 | 21 ± 3 |
| Creatinine (mg/dl) | 0.2 ± 0.13 | 0.2 ± 0.08 | 0.2 ± 0.14 | 0.2 ± 0.12 |

Table S3. Antibody Source and Use

| Name/Antigen | Company | Catalog # | Dilution | Western Blot (WB) or  Immunohistochemistry (IHC) |
| --- | --- | --- | --- | --- |
| Actin | Santa Cruz | Sc-47778 | 1:2000 | WB |
| Dinitrophenylhydrazine | EMD-Millipore | S7150 | 1:150 | WB |
| GAPDH | Cell Signaling | 2118 | 1:2000 | WB |
| JNK | Cell Signaling | 9252 | 1:1000 | WB |
| Nitrotyrosine | Millipore-Sigma | 06-284 | 1:300 | IHC |
| 8-OH-deoxyguanosine | Abcam | ab26842 | 1:100 | IHC |
| Phospho-JNK(Thr^183^/Tyr^185^) | Cell Signaling | 9251 | 1:1000 | WB |

**Video: Cbi(II) Reaction with Superoxide**

To a 20 µM solution of Cbi(II) in potassium phosphate, pH 7.1 was added KO_2_ to a final concentration of 62.5 µM (the KO_2_ was dissolved in DMSO). The video was recorded at 30 frames per second. For comparison, the wells on either side of the test well contain Cbi(II) on the left and Cbi(III) on the right. The experiment was conducted in a 96 well tissue culture plate, and was repeated several times with identical results.

**REFERENCES**

1. Zielonka, J., Vasquez-Vivar, J., and Kalyanaraman, B. (2008) Detection of 2-hydroxyethidium in cellular systems: a unique marker product of superoxide and hydroethidine. *Nat Protoc* **3**, 8-21

2 Stutelberg, M. W., Dzisam, J. K., Monteil, A. R., Petrikovics, I., Boss, G. R., Patterson, S. E., Rockwood, G. A., and Logue, B. A. (2016) Simultaneous determination of 3-mercaptopyruvate and cobinamide in plasma by liquid chromatography-tandem mass spectrometry. *J Chromatogr B Analyt Technol Biomed Life Sci* **1008**, 181-188

3. Villamena FA, Zweier, J.L. Superoxide radical trapping and spin adduct decay of 5-*tert*-butoxycarbonyl-5-methyl-1-pyrroline *N*-oxide (BocMPO ): kinetics and theoretical analysis. *J Chem Soc, Perkin Trans.* 2002;2:1340-4.
